# MetaOmGraph: a workbench for interactive exploratory data analysis of large expression datasets

**DOI:** 10.1093/nar/gkz1209

**Published:** 2020-01-20

**Authors:** Urminder Singh, Manhoi Hur, Karin Dorman, Eve Syrkin Wurtele

**Affiliations:** 1 Bioinformatics and Computational Biology Program, Iowa State University, Ames, IA 50011, USA; 2 Center for Metabolic Biology, Iowa State University, Ames, IA 50011, USA; 3 Department of Genetics Development and Cell Biology, Iowa State University, Ames, IA 50011, USA; 4 Department of Statistics, Iowa State University, Ames, IA 50011, USA

## Abstract

The diverse and growing omics data in public domains provide researchers with tremendous opportunity to extract hidden, yet undiscovered, knowledge. However, the vast majority of archived data remain unused. Here, we present MetaOmGraph (MOG), a free, open-source, standalone software for exploratory analysis of massive datasets. Researchers, without coding, can interactively visualize and evaluate data in the context of its metadata, honing-in on groups of samples or genes based on attributes such as expression values, statistical associations, metadata terms and ontology annotations. Interaction with data is easy via interactive visualizations such as line charts, box plots, scatter plots, histograms and volcano plots. Statistical analyses include co-expression analysis, differential expression analysis and differential correlation analysis, with significance tests. Researchers can send data subsets to R for additional analyses. Multithreading and indexing enable efficient big data analysis. A researcher can create new MOG projects from any numerical data; or explore an existing MOG project. MOG projects, with history of explorations, can be saved and shared. We illustrate MOG by case studies of large curated datasets from human cancer RNA-Seq, where we identify novel putative biomarker genes in different tumors, and microarray and metabolomics data from *Arabidopsis thaliana*. MOG executable and code: http://metnetweb.gdcb.iastate.edu/ and https://github.com/urmi-21/MetaOmGraph/.

## INTRODUCTION

Petabytes of raw and processed data generated with microarray, RNA-Seq (bulk and single-cell) and mass spectrometry for small molecules and proteins are available through public data repositories (1–4). These data represent multiple species, organs, genotypes and conditions; some are the results of groundbreaking research. Buried in these data are biological relationships among molecules that have not yet been explored. Integrative analysis of data from the multiple studies representing diverse biological conditions is the key to fully exploit these vast data resources for scientific discovery (5,6). Such analysis allows efficient reuse and recycling of these available data and metadata (1,5,7). Higher statistical power can be attained with bigger datasets, and the wide variety of biological conditions can reveal the complex regulatory structure of genes. Yet, despite the availability of such vast data resources, most bioinformatic studies use only a limited amount of the available data.

A common goal of analyzing omics data is to infer functional roles of particular features (genes, proteins, metabolites or other biomolecules) by investigating co-expression and differential expression patterns. A wide variety of R-based (8) tools can provide specific analyses (9–12). Such tools are based upon rigorous statistical frameworks and produce accurate results when the model assumptions hold. Several tools avoid the need to code by providing ‘shiny’ interfaces (13) to various subsets of R’s functionalities (14–20). Such R tools based on the ‘shiny’ interface have the general limitations that they are not well suited for very large datasets and can have limited interactivity.

Increasing the usability of the vast data resources by enabling efficient exploratory analysis would provide a tremendous opportunity to probe the expression of transcripts, genes, proteins, metabolites and other features across a variety of different conditions. Such exploration can generate novel hypotheses for experimentation, and hence improving the fundamental understanding of the function of genes, proteins and their roles in complex biological networks (6–7,21–25).

At present, there are very limited options for researchers to interact with expression datasets using the fundamental principles of exploratory data analysis (26). Exploratory data analysis is a technique to gain insight into a dataset, often using graphical methods which can reveal complex associations, patterns or anomalies within data at different resolutions. By adding interactivity for visualizations and statistical analyses, researchers with little or no programming experience are able to directly explore the underlying, often complex and multidimensional, data themselves. Researchers in diverse domains (e.g. experts in Parkinson’s disease, malaria or nitrogen metabolism) can mine and re-mine the same data, extracting information and deriving testable hypotheses pertinent to their particular areas of expertise. These hypotheses can inform the design of new laboratory experiments. Being able to explore and interact with data becomes even more critical as datasets become larger. The information content inherent in the vast stores of public data is enormous. Due to the sheer size and complexity of such big data, there is a pressing requirement for effective interactive analysis and visualization tools (27,28).

In this paper, we present MetaOmGraph (MOG), a Java software, to interactively explore and visualize large expression datasets. MOG overcomes the challenges posed by the size and complexity of big datasets by efficient handling of the data files. Further, by incorporating metadata, MOG adds extra dimensions to the analyses and provides flexibility in data exploration. At any stage of the analysis, a researcher can save her/his progress. Saved MOG projects can be shared, reused and included in publications. MOG is user-centered software, designed for exploring diverse types of numerical data and their metadata, but specialized for expression data.

## MATERIALS AND METHODS

### Overview

**Figure 1.**
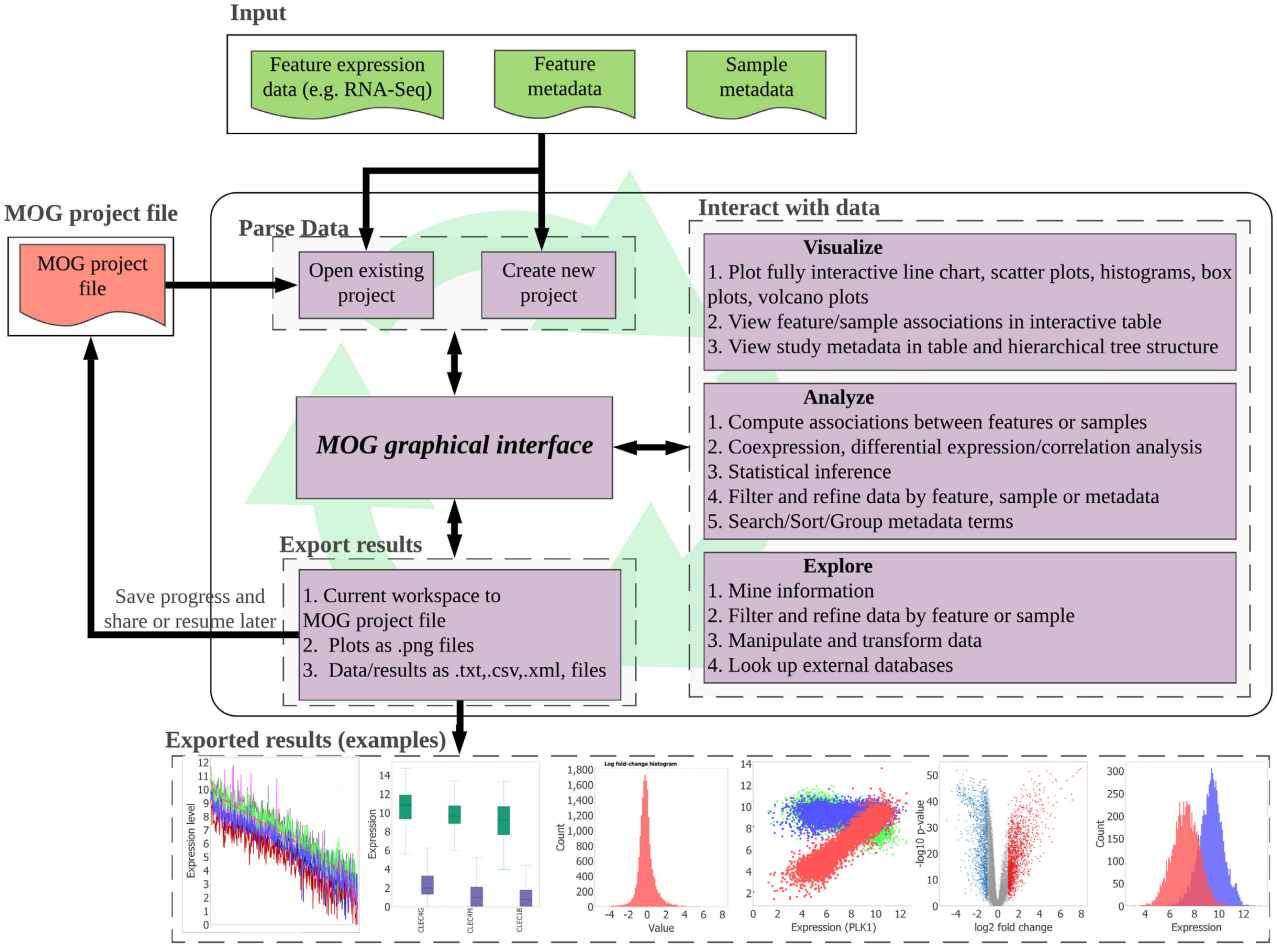
An overview of MOG’s modules. All functionality is accessed through MOG’s GUI. First, the researcher selects an existing MOG project or creates a new MOG project (.mog) with input data files. Once the project is open in MOG, the workflow is non-linear. The GUI enables interactive exploration of data through a choice of statistical analyses and data visualizations. The researcher can export visualizations and results throughout the analysis, and can save her/his feature lists and statistical analyses in the MOG project file for future exploration. Saved MOG projects can be shared and further analyzed by new researchers.

MOG is an interactive software that can run on any operating system capable of running Java (Linux, Mac and Windows). MOG’s Graphical User Interface (GUI) is the central component through which all the functionality is accessed (Figure 1). Access to MOG is easy. MOG is a standalone program and runs on the researcher’s computer; thus, the researcher does not need to rely on internet accessibility for computations, and is not slowed down by the data transfer latency. Furthermore, the data in a researcher’s project is secure, remaining on the researcher’s computer, particularly important for confidential data such as human RNA-Seq.

#### Interactive data exploration

MOG displays all the data in interactive tables and trees, providing a flexible and structured view of the data. The user can interactively filter or select data for analysis. This ability is particularly important for aggregated datasets, as users may wish to split data into groups of studies, treatments or organs. A novel aspect of MOG is its capability of producing *interactive* visualizations. The researcher can visualize data via line charts, histograms, box plots, volcano plots, scatter plots and bar charts, each of which is programmed to allow real-time interaction with the data and the metadata. Users can group, sort, filter, change colors and shapes, zoom and pan interactively, via the GUI. At any point in the exploration, the researcher can look-up external databases: GeneCards (29), Ensembl (30), EnsemblPlants (31), RefSeq (32), TAIR (33) and ATGeneSearch (http://metnetweb.gdcb.iastate.edu/MetNet_atGeneSearch.htm) for additional information about the genomic features in the dataset. Researchers can also easily access SRA and GEO databases using the accessions present in the study metadata.

#### Efficient, multithreaded and robust

A key advantage of MOG is its minimal memory usage, enabling datasets to be analyzed that are too large for other available tools. Researchers with a laptop/desktop computer can easily run MOG with data files containing thousands of samples and fifty thousands of transcripts. MOG achieves computational efficiency via two complementary approaches. First, MOG indexes the data file, rather than storing the whole data in main memory. This enables MOG to work with very large files using a minimal amount of memory. Second, MOG speeds up the computations using multithreading, optimizing the use of multi-core processors. MOG is robust and can cope with most of the errors and exceptions (such as missing values or forbidden characters) that can occur when handling diverse data types. Bug reports can be submitted with a single click, if encountered.

#### Data-type agnostic

Although specifically created for the analysis of omics data, which is the focus of this paper, MOG is designed to be flexible enough to generally handle numerical data. A user can supplement a MOG project with any type of metadata about the features, and about the studies. Thus, a MOG user can interactively analyze and visualize voluminous data on any topic. For example, a user could create a project on: transmission of mosquito-borne infectious diseases world-wide; public tax return data for world leaders over the past 40 years; daily sales at Dimo’s Pizza over 5 years; player statistics across all Women’s National Basketball Association (WNBA) teams; climate history and projections since 1900.

#### Leverage of third party Java libraries

In addition to the functionality we have programmed into MOG, MOG borrows some functionality from freely available and extensively tested third-party Java libraries (JFreeChart, Apache Commons Math, Nitrite and JDOM). We have combined these to create a highly modular system that is amendable to changes and extensions and developers can easily implement new statistical analyses and visualizations in the future. MOG is an open source project and we plan to expand and develop it further through community driven efforts. Information on how to contribute to MOG, and who to contact with further questions, is provided at https://github.com/urmi-21/MetaOmGraph/blob/master/CONTRIBUTING.md.

#### Interface to R

Based on the utility and popularity of R for data analysis, we have implemented a GUI to facilitate execution of R scripts through MOG. MOG’s GUI enables a user to interactively select or filter data using MOG; these data are then passed to R. This avoids the need to constantly write new R code to specify different genes and samples for analyses. For example, a user can write an R script for hierarchical clustering of genes based on the expression levels, interactively select or filter data using MOG, and execute the R script. More details on how to use MOG for executing R scripts are provided in the user manual available from (https://github.com/urmi-21/MetaOmGraph/tree/master/manual).

### Creating a new MOG project or using an existing one

A user can quickly create a new MOG project using two delimited files: (i) a file with unique identifiers (IDs) for each feature (e.g. gene), metadata about that feature and numerical data quantifying each feature across multiple conditions (e.g. multiple samples and studies), and (ii) a file containing unique identifiers for each sample and metadata about the samples and studies in the datafile. These are virtually combined by MOG, using the unique identifier in each file (Supplementary Figure S1). Selecting appropriate methods for data normalization, batch correction and vetting are important considerations for a user when creating a new project (Supplementary File 1).

New MOG projects, as well as those from well-vetted datasets, including the human and *Arabidopsis thaliana* datasets described herein, can be re-opened, analyzed, modified or shared. Ongoing exploration results, such as correlations, lists and other interactive analyses, can be saved in any MOG project, regardless of whether it was obtained from our website or created from custom data.

### Detecting statistical association within data

Measures of statistical association between a pair of features in a dataset quantify the similarity in their expression patterns across the samples that comprise that dataset (34–37). Genes with significant statistical association may participate in common biological processes and pathways (23,34,38). Genes with significant association only under specific conditions may reveal their functional significance under those conditions (39,40).

MOG provides the researcher with several statistical measures to estimate associations/co-expression among the features. It can also compute association between samples, which reflects similarity between the samples. Choosing appropriate statistical measures and interpretations for each dataset is left to the user.

#### Correlation, mutual information and relatedness

We have incorporated four key methods that measure association among pairs of features. Each has its own advantages and disadvantages, depending on the types of relationships the researcher wishes to detect, and the characteristics of the dataset being explored.

MOG can compute pairwise Pearson and Spearman correlation for pairs of selected features across all samples or conversely, between selected samples across all features. The Pearson correlation coefficient measures the extent of a linear relationship between two random variables, *X* and *Y*, whereas, the Spearman correlation coefficient measures monotonic relationships between the two variables. Both excel at detecting linear relationships, however, Spearman is less sensitive to outliers (41). Pearson and Spearman correlations are often used to find co-expressed genes and generate matrices used for inferring gene expression networks (23,35,39).

MOG also computes pairwise mutual information (MI) between selected features across samples. MI quantifies the amount of information shared between two random variables. Let (*X*, *Y*) be a pair of discrete random variables over the space $\mathcal {X} \times \mathcal {Y}$. Then, the MI for *X* and *Y* is defined as:

$$\begin{equation*} I(X;Y)=\sum _{y\in \mathcal {Y}} \sum _{x\in \mathcal {X}} p(x,y) \log \left( \frac{p(x,y)}{p(x)p(y)} \right), \end{equation*}$$

where, *p*(*x*, *y*) is the joint probability mass function of *X* and *Y*, and *p*(*x*) and *p*(*y*) are the marginal probability mass functions for *X* and *Y*, respectively. Compared to correlation measures, MI is a more general approach that can detect complex, non-linear associations. The interpretation of the MI value is different than that of correlation values: an MI value of zero, *I*(*X*; *Y*) = 0, implies statistical independence of *X* and *Y*, whereas a correlation value of zero need not imply statistical independence (41). MI has been applied to detect non-linear associations in gene expression datasets (25,42–44). MOG computes MI using B-splines density estimation, as described in Daub *et al.* (42).

MOG can also determine the context likelihood of relatedness (CLR) (37). CLR determinations aim to identify biologically relevant associations by discounting features (e.g. genes) that have promiscuous associations. Specifically, the CLR compares the MI value between each pair of features to the background distribution of MI values that include either of these features (37).

#### Meta-analysis of correlation coefficients

MOG can perform meta-analysis of Pearson correlations. Studies using microarray data showed that meta-analysis and analysis of pooled normalized samples each bring out meaningful, but different, relationships among genes (24). For meta-analysis of correlation coefficients, MOG calculates a weighted average of the individual Pearson correlation coefficients computed from each study. The weights are proportional to the sample size, i.e. correlations estimated from larger studies are more trusted (45,46). Meta-analysis can be useful when multiple studies run a similar experiment (e.g. effect of heat-stress on *A. thaliana*), but may not control ancillary sources of variation (e.g. coverage variation in RNA-Seq data). MOG provides a choice between a fixed effects model (FEM) or a random effects model (REM) (45,46) for the meta-analysis. The FEM combines the estimated effects by assuming that all studies probe the same correlation in the same population, i.e. studies are homogeneous. In contrast, the REM allows studies to be heterogeneous, with additional, uncontrolled sources of variation (45,46). The FEM does not account for all heterogeneities, thus the researcher should choose a model and interpret the results with appropriate caution.

### Differential expression between groups

Determining differentially expressed features from aggregated datasets provides direction for further data exploration. In MOG, we have incorporated several popular statistical methods to evaluate differential expression between two groups of samples. For analysis of groups with independent samples, we have implemented: Mann–Whitney U test (a non-parametric test that makes no assumptions about data distribution); Student’s *t*-test (assumes equal variance and normally distributed data); Welch’s *t*-test (does not assume equal variance, assumes a normal distribution of data); and a permutation test (makes no assumptions about data distribution; computes null distribution empirically using the data). For analysis of groups with paired samples, we have implemented: a Paired *t*-test (assumes normal distribution of data); a Wilcoxon signed-rank test (a non-parametric test; no assumption of data distribution); and a permutation test for paired samples (makes no assumptions about data distribution but computes null distribution empirically using the data).

MOG’s methods to identify differentially expressed genes are general statistical methods which are designed for large sample sizes (30 or more samples for gene expression data). Computation of these methods via MOG permits interactivity, which promotes data exploration. A limitation of the interactive differential expression analysis methods implemented in MOG is that they are designed for large sample sizes and use normalized data as input. For smaller sample sizes, a user can apply specialized model-based methods, accessible through R, to infer differentially expressed genes in RNA-Seq or microarray datasets. For example, methods like edgeR (12), DESeq2 (11) and limma (10) require raw counts as input and can provide more reliable differential expression analysis (47) for smaller sample sizes. Tools like ideal (20) and DEBrowser (19) provide interactive interface for accessing these popular differential expression analysis methods (10–12).

### Differential correlation between groups

Features whose correlation with other features is significantly different only under particular environmental, genetic or developmental conditions are designated as differentially correlated. Such *shifting* biological interactions among these genes or their regulators (40,48) reflect the context-dependency of gene expression.

MOG can find the features whose Pearson correlation to a user-selected feature differs significantly between two groups of samples. To do this, MOG applies a Fisher transformation (49) and performs a hypothesis test for equality of Pearson correlation coefficients from the two groups. (The difference of the two Fisher transformed Pearson correlation coefficients follows a normal distribution (40)). The researcher can choose to conduct a test for statistical significance on the Fisher transformed Pearson correlation coefficients or on the raw Pearson correlation coefficients.

### Statistical significance determinations

For each statistical test, MOG provides a non-parametric option (a permutation test) and parametric options (calculations under distributional assumptions) to estimate *P*-values.

Empirical *P*-values are calculated by a permutation test that estimates the null distribution of a test statistic by randomly permuting the labels of the observed data points (assuming that the labels are exchangeable under the null hypothesis) (50). Because permutation tests do not rely on any data distribution, they are applicable even if parametric assumptions are not met. More permutations yield more precise estimates of the null distribution and *P*-values, but at the cost of longer computation times. MOG accelerates computation of permutation tests by multithreading, and processing the permuted datasets in parallel (Supplementary File 1).

MOG provides three popular parametric methods to adjust the *P*-values for multiple comparisons: the Bonferroni method (51), the Holm method (52) and the Benjamini–Hochberg (BH) method (53). Bonferroni and Holm methods are applied to control the family-wise error rate (FWER), whereas the BH method controls the false discovery rate (FDR). Controlling the FWER limits the total number of false positives; the Holm method is less conservative as compared to the Bonferroni method. In contrast, controlling the FDR controls the proportion of false positives among the significant tests.

### Datasets

To create case-studies with MOG, we assembled MOG projects based on three technical platforms.

#### Human cancer RNA-Seq dataset (7142 samples)

We created a new MOG project based on the well-vetted dataset from Wang *et al.*, (21). This dataset combines RNA-Seq data from The Cancer Genome Atlas (TCGA, tumor and non-tumor samples) (https://cancergenome.nih.gov/) and Genotype Tissue Expression (GTEX, non-tumor samples) (54).

To create the MOG project, we excluded from the dataset any organ types in which the number of tumor or non-tumor samples was <30. To ensure statistical independence among the samples, we removed all non-tumor samples from TCGA and included only one TCGA tumor-sample per patient (Supplementary File 2). We also excluded an outlier sample with very low expression values, based on a preliminary exploration of sample replicates using MOG (See ‘Results’ section).

We then compiled metadata for the studies/samples and for the genes and integrated this metadata into the dataset. We downloaded the study and sample metadata (TCGA metadata from TCGAbiolinks (55); GTEX metadata from GTEX’s website (https://gtexportal.org/home)). We were unable to locate metadata for 17 of the TCGA samples and excluded these samples from the dataset (Supplementary File 2). We extracted metadata about the genes from the HGNC (https://www.genenames.org/), NCBI Gene (https://www.ncbi.nlm.nih.gov/gene), Ensembl (30), Cancer Gene Census (56) and OMIM (57) databases and added these information to the gene metadata in our dataset. We also eliminated the 1870 genes that were not reported for all studies resulting in a dataset, called herein, ‘*Hu-cancer-RNASeq-dataset*’.

We generated the MOG project (*Hu-Cancer-18212-7412-RNASeq.mog*) from the *Hu-cancer-RNASeq-dataset* and its metadata. The MOG project contains expression values for 18 212 genes, 30 fields of metadata detailing each gene, across 7142 samples representing 14 different cancer types and associated non-tumor tissues (Table 1); it also has 23 fields of metadata describing each study and sample in the dataset. We used MOG to *log*_2_ transform the data for subsequent analyses.

**Table 1.** Tumor and non-tumor samples in the *Hu-cancer-RNASeq-dataset* and the number of *up*regulated and *down*regulated genes in each tumor type with respect to the corresponding normal samples, as calculated by MOG

TCGA disease	GTEX	#TCGA	#GTEX	Total	#Up	#Down
	organ	samples	samples			
Breast invasive carcinoma (BRCA)	Breast	965	89	1054	1093	2827
Colon adenocarcinoma (COAD)	Colon	277	339	616	1401	3036
Esophageal carcinoma (ESCA)	Esophagus	182	659	841	1989	2229
Kidney Chromophobe (KICH)	Kidney	60	32	92	986	4214
Kidney renal clear cell carcinoma (KIRC)	Kidney	470	32	502	1877	2263
Kidney renal papillary cell carcinoma (KIRP)	Kidney	236	32	268	1152	2737
Liver hepatocellular carcinoma (LIHC)	Liver	295	115	410	1527	1485
Lung adenocarcinoma (LUAD)	Lung	491	313	804	1361	2753
Lung squamous cell carcinoma (LUSC)	Lung	486	313	799	2210	3734
Prostate adenocarcinoma (PRAD)	Prostate	426	106	532	577	1633
Stomach adenocarcinoma (STAD)	Stomach	380	192	572	1527	1631
Thyroid carcinoma (THCA)	Thyroid	441	318	759	993	1525
Uterine Corpus Endometrial Carcinoma (UCEC)	Uterus	141	82	223	2135	3250
Uterine Carcinosarcoma (UCS)	Uterus	47	82	129	2419	2491

#### *A. thaliana* microarray dataset (424 samples)

We created a new MOG project, *AT-Affy-22746-424-microarray.mog*, based on the *A. thaliana* curated microarray dataset (*‘AT-microarray-dataset’*) from Mentzen and Wurtele (23). This dataset had been compiled using 963 Affymetrix ATH1 chips with 22 746 probes from 70 diverse studies encompassing different conditions of development, stress, genotype and environment. All chips in the dataset were individually normalized and scaled to a common mean using MAS 5.0 algorithm. Only chips with good quality biological replicates were kept and all the biological replicates were averaged to yield 424 samples. At last, median absolute deviation (MAD)-based normalization (58) was applied to the data. We compiled new metadata for the genes from TAIR gene annotations (33) and added phylostratal inferences (59). The sample metadata were obtained from Mentzen and Wurtele (23).

#### *A. thaliana* metabolomics GC-MS dataset (656 samples)

The small molecule composition (metabolomics) data that we used to create a MOG project were from 656 GC-MS samples describing the effect of 50 knock out (or knock down) mutations of genes of mostly unknown functions on the accumulation of metabolites in *A. thaliana* (60) (called herein, *‘AT-metab-dataset’*). We downloaded these data from the Plant/Eukaryotic and Microbial Resource (PMR) (61). We created the MOG project *AT-Mutation-242-656-metab.mog* with this dataset.

## RESULTS

We illustrate MOG’s usability and flexibility by exploring three diverse datasets from different perspectives. The statistical analyses and visualizations shown were generated exclusively using MOG. Often, our exploration led us to conclusions consistent with prior experimental or *in silico* results. In other cases, the exploration led us to completely novel predictions that could be tested experimentally.

### Preliminary exploration of the *Hu-cancer-RNASeq-dataset*

Determining that a dataset is valid, properly normalized and free of batch effects is a critical preliminary step in the analysis. To verify that samples from similar biological conditions exhibit similar expression patterns for all the genes, we used MOG to compute pairwise Pearson correlations among samples from the same biological condition (tumor/non-tumor and organ type). All the samples had high Pearson correlations (>0.70) with others taken from the same organ and tumor status, except one sample from lung adenocarcinoma (LUAD), which we removed from the dataset (Additional File 1).

We visualized the distribution of Pearson correlation values for non-tumor samples. For homogeneous samples, such distributions should appear unimodal. However, several organs show multimodal distributions (Supplementary Figure S2). This finding led us to conjecture that samples might have been taken from different anatomical sites within these organs. By exploring further with MOG, we were able to identify additional metadata on sub-locations in the colon and esophagus that support this conjecture (Supplementary Figure S2). However, the stomach sample metadata does not further specify location (or any other obvious factor, such as gender, race or age) that might distinguish subgroups of samples. Because the stomach samples are of several distinct types, a researcher might want to consider analyzing them as such.

### Using MOG to identify a catalog of differentially expressed genes in cancers

We wanted to identify *key genes* that are regulated by, or implicated in, the molecular and cellular processes driving cancer, and to further explore the processes in which these genes are involved. For this task, we used MOG first to identify the differentially expressed genes in samples from each tumor type versus corresponding non-tumor samples, and then to examine the expression patterns of these genes. We define a gene as differentially expressed between two groups if it meets each of the following criteria:

- Estimated fold change in expression of 2-fold or more (log  fold change, |*logFC*| ≥ 1 where *logFC* is calculated as in limma (10).)
- Mann–Whitney U test, on the log_2_ transformed data, is significant between the two groups (BH corrected *P*-value <10^−3^)

In each type of cancer in the *Hu-cancer-RNASeq-dataset*, between 2000–5000 of the 18 212 genes are differentially expressed (Table 1 and Supplementary File 3). Thirty-five of these genes are consistently differentially expressed in all of the cancers (Table 2).

**Table 2.** MOG identifies 35 genes as differentially expressed in all of the 14 tumor types

Upregulated in each cancer	Downregulated in each cancer
BIRC5, BUB1, CDC45	ADH1B, C7, CHRDL1
CDKN2A, CENPF, DLGAP5	CMTM5, DCN, DES
FAM111B, KIF4A, KIF20A	DPT, GPM6A, GSTM5
MELK, MKI67, PBK	HPD, HSPB6, MRGPRF
PKMYT1, TOP2A, TPX2	NKAPL, PEG3, PI16
UBE2C	PTGDS, SCN7A, TCEAL2
	TGFBR3

(Mann–Whitney U test, |FC| ≥ 2, BH corrected *P*-value <10^−3^).

**Figure 2.**
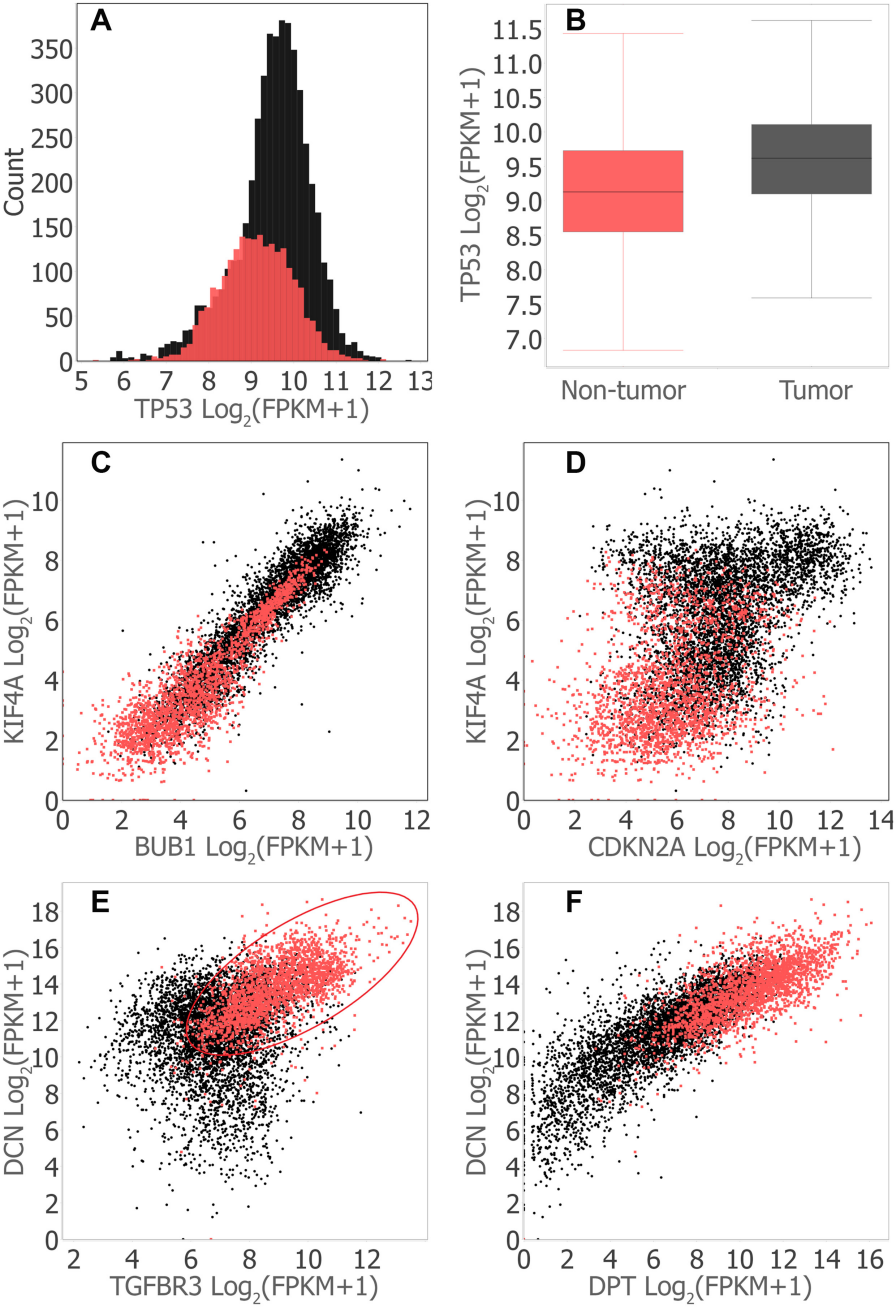
MOG visualizations of expression of selected genes across all tumor types and non-tumor samples. Tumor samples, black dots; non-tumor samples, red dots. Correlations and differential expression analyses were performed using MOG (Mann–Whitney U test, |*FC*| ≥ 2, BH corrected *P*-value <10^-3^). Plots were generated in MOG by interactively splitting the gene expression data into the categories ‘tumor’ and ‘non-tumor’ using the sample metadata. (**A**) Histogram showing the distribution of Tumor Protein P53 (TP53) expression (number of bins set to 50). (**B**) Box plot summarizing the expression of TP53 over all tumor versus all non-tumor samples. The horizontal line inside the box represents the median log expression, which is 9.1 for non-tumor samples and 9.6 for tumor samples. (**C**) Scatter plot visualizing co-expression of mitotic checkpoint serine/threonine kinase (BUB1) and kinesin family member 4A (KIF4A) (both are upregulated across all tumor types). (Spearman correlation=0.92 in non-tumor samples; Spearman correlation=0.84 in tumor samples; Spearman correlation=0.94 over both tumor and non-tumor samples). (**D**) Scatter plot visualizing co-expression of genes cyclin dependent kinase inhibitor 2A (CDKN2A) and KIF4A. Both are upregulated across all tumor types, but they are are not co-expressed. (**E**) Scatter plots visualizing transforming growth factor beta receptor3 (TGFBR3), which has a complex role as regulator of angiogenesis (117), decorin (DCN), autophagy, mitophagy and embryonic cell development including endovascular differentiation (118). TGFBR3 and DCN are downregulated across all tumor types and are co-expressed in non-tumor samples (Spearman correlation=0.64) but not in tumor cells (Spearman correlation=0.14). The co-expression of TGFBR3 and DCN in only the non-tumor samples suggests that the processes in which each gene participates are associated under normal conditions; the loss of this association in tumors is consistent with a hypothesis that an imbalance, or factors that cause that imbalance, may further contribute to the etiology of cancer. (**F**) Scatter plot visualizing co-expression of genes dermatopontin (DPT) and DCN Spearman correlations are 0.82 (tumor samples), 0.69 (non-tumor samples) and 0.84 (combined samples) (Both gene are downregulated across all tumor types.)

Several genes that are deeply implicated in cancer are not differentially expressed in any of the tumor types we analyzed. One example is tumor suppressor protein 53 (TP53) (Figure 2 A and B). (TP53 is differentially expressed in colorectal tumors (62); colorectal tumors are not included in the *Hu-cancer-RNAseq-dataset*).

Fifteen of the 16 genes upregulated across all tumor types are co-expressed across the tumor samples, across the non-tumor samples and across the combined tumor plus non-tumor samples (Figure 2 C and Supplementary File 4). Cyclin dependent kinase inhibitor (CDKN2A) is an outlier (Spearman correlation < 0.50) (Figure 2 D and Supplementary File 4). This co-expression might imply that these 15 genes function together as a module in both tumor and non-tumor cells.

In contrast, there is no co-expression cluster among the 19 genes that are downregulated across all cancer types; 62 individual gene pairs are correlated across all the samples (Spearman correlation ≥ 0.60) (Supplementary File 4). Expression of seven of these gene pairs is strongly correlated only among tumor samples but is not correlated among non-tumor samples; conversely, 18 gene pairs are strongly correlated among non-tumor samples but not among the tumor samples (e.g. Figure 2E)—this finding indicates a context-dependent coordination of these gene pairs. Four gene pairs are strongly correlated among both tumor and in non-tumor samples (e.g Figure 2F).

#### Functional analysis of differentially expressed genes

To determine whether the genes that are differentially expressed in cancers are involved in known biological processes, we performed gene ontology (GO) enrichment analysis using GO::TermFinder (63) and Revigo (64) on the genes that are upregulated, downregulated or not significantly changed across all the cancer types. Consistent with the behavior of cancer cells, upregulated genes are significantly enriched in GO terms related to cell proliferation: cell cycle, cell division, organelle organization, regulation of cellular component organization and regulation of cell cycle (Supplementary File 4 and Figure S3). The 5784 genes that did not change expression were enriched in GO terms RNA processing, mRNA metabolic process, nucleic acid metabolic process and gene expression (Supplementary Figure S4 and File 4). The downregulated genes show no significant GO term enrichment.

### Using MOG for gene-level exploration

With the aim to use MOG from the vantage point of an individual gene, we selected the glypican 3 (GPC3) gene as an interesting candidate for a case study. GPC3, encoding a glycosylphosphatidylinositol-linked heparan sulfate proteoglycan, is located on the X chromosome and has been implicated as a critical regulator of tissue growth and morphogenesis (65). GPC3 inhibits cell proliferation and hedgehog signaling during embryonic development (66). In tumors, GPC3’s role is complex and not well understood. It can promote or inhibit cell growth depending on the cancer type (67,68). Mutations in GPC3 have been linked to Wilms tumor as well as Simpson-Golabi-Behmel syndrome (SGBS) (69,70).

#### GPC3 Expression patterns

**Figure 3.**
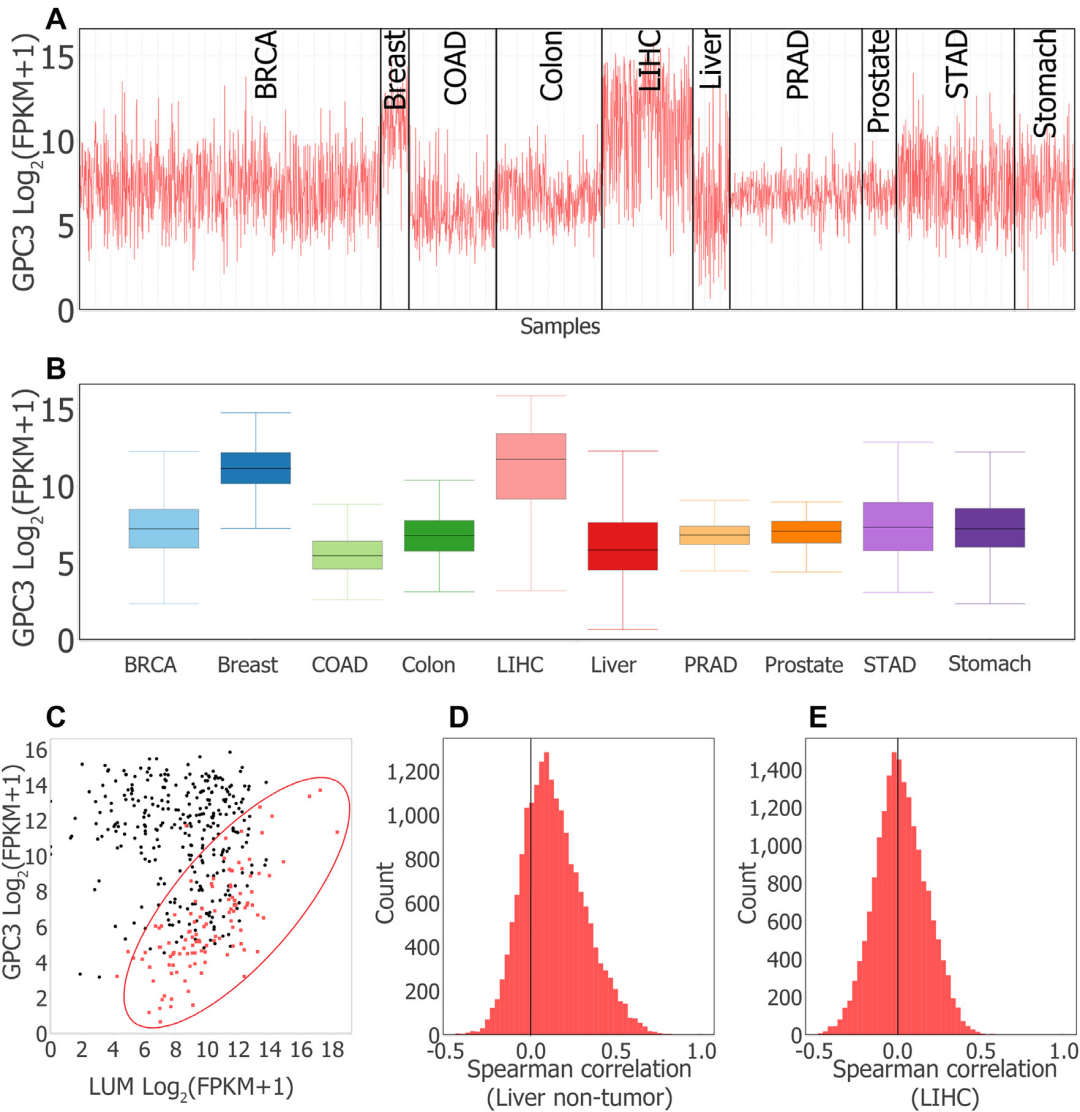
MOG visualizations of glypican 3 (GPC3) expression pattern in tumor and non-tumor organs. (**A**) Line chart generated by interactively filtering by study metadata to retain 3184 samples from 5 tumor types and corresponding non-tumor organs, and grouping the chart by organ/tumor type. (**B**) Box plot summary of data in (A). Generated by interactively splitting box plot according to organ/tumor type. (**C**) Scatter plot showing co-expression of GPC3 and Lumican (LUM) in liver non-tumor and LIHC samples. In non-tumor liver (red), GPC3 and LUM expression are strongly correlated (Spearman correlation ≤ 0.7). In LIHC samples (black), GPC3 and LUM expression show no association (Spearman correlation = −0.1). (**D** and **E**) Histograms of distribution of Spearman correlation coefficients of expression of GPC3 with all other genes. Non-tumor liver samples (D), LIHC samples (E). The longer right tail of non-tumor liver samples indicates Spearman correlation coefficients of GPC3 expression with selected genes are higher in non-tumor than LIHC samples.

We explored expression patterns of GPC3 with regards to the 14 tumor types. Differential expression of GPC3 in non-tumor versus tumor samples varies by organ. GPC3 expression is 30-fold higher in the LIHC samples than in the non-tumor liver samples, and 8-fold higher in the UCS samples compared to the non-tumor uterus samples (Supplementary File 5). In contrast, GPC3 is downregulated in nine tumor types (BRCA, COAD, ESCA, KIRC, KIRP, LUAD, LUSC, THCA and UCEC) and unchanged in three tumor types (KICH, STAD and PRAD) (Figure 3A and B; Supplementary File 5).

These results are consistent with targeted studies of liver, breast and lung tumors. GPC3 expression is upregulated in liver cancer (67,71–72), and has been suggested as a diagnostic biomarker and as a potential target for cancer immunotherapy in hepatocellular carcinoma (71–73). GPC3 is downregulated in breast (74), lung (75) and ovarian cancers (76), and it may act as a tumor suppressor in lung and renal cancer (76,77).

#### GPC3 Co-expression patterns

We then investigated co-expression patterns of GPC3 in the tumor and non-tumor tissues from different organs (Additional File 3). GPC3 co-expression patterns differ between tumor and non-tumor samples according to the organ sampled (Figure 3C), moreover, the genes whose expression is correlated with GPC3 are distinct according to organ types, all reflecting the complex role of this gene (Supplementary File 5). For example, 4219 genes are co-expressed with GPC3 in non-tumor esophagus samples, whereas no gene is co-expressed with GPC3 in non-tumor samples from prostate and stomach (Supplementary File 5). Co-expressed genes also differed according to whether disease was present. For seven organs, fewer genes were co-expressed with GPC3 in tumor samples than in non-tumor samples (Supplementary File 5). For example, 192 genes were co-expressed with GPC3 in non-tumor liver samples, whereas no genes were significantly co-expressed with GPC3 in LIHC tumor samples (Figure 3D and E).

We analyzed GO term enrichment for those organs with more than 10 GPC3-co-expressed genes: colon, esophagus, kidney and liver. The term cell adhesion is enriched in GPC3-co-expressed genes from colon, esophagus, kidney and liver. The terms cell development, extracellular matrix organization and multicellular organism development are enriched among GPC3-co-expressed genes in colon, esophagus and kidney. Other GO terms are over-represented in a organ-specific manner (Supplementary File 5).

#### GPC3-associated clusters in tumor versus non-tumor samples from liver

To further explore potential interactions of GPC3 with other genes, we used MOG to build two gene co-expression networks from the 3012 genes that are differentially expressed in LIHC—one network from non-tumor liver samples, and a second from LIHC samples (Additional File 3). Then, we imported each network into Cytoscape (78) and identified the tightly connected modules using MCODE (79).

In the network built from non-tumor liver samples, MCODE ranked the GPC3-containing cluster second most significant (73 nodes (genes); MCODE score 30.7). GPC3 was directly connected with 21 genes in this cluster (Supplementary Figure S5), which is most enriched in GO terms: sulfur compound catabolic process, aminoglycan catabolic process and extracellular matrix organization (Supplementary Figure S6 and File 5).

In contrast, in the LIHC samples, GPC3 was not significantly co-expressed with any other genes, and thus was absent from the entire LIHC network. However, the LIHC network does contain a module with 114 genes (MCODE score 94.3), 33 of which are in the GPC3-containing cluster identified from the non-tumor network (17 of these genes are directly connected with GPC3 in the non-tumor network) (Supplementary Figure S5). This cluster is enriched in GO terms: extracellular matrix organization, blood vessel development and vasculature development (Supplementary File 5 and Figure S7).

### Stage-wise analysis of *Hu-cancer-RNASeq-dataset*

#### Identifying new candidate biomarkers for cancers

To identify potential biomarkers for tumors, we used MOG to distinguish genes whose expression is associated with the disease progression. We used MOG to separate samples by organ, and then by early stage (stage I or stage II) and late stage (stage III and later), based on the study metadata. At last, we performed a Mann–Whitney U test on those genes that are upregulated in tumor versus non-tumor samples (Supplementary File 3) to reveal the genes that are upregulated in late stage compared to early stage (expression increase 2-fold or more, and BH corrected *P*-value < 0.05). These genes have increasing expression with cancer progression. We similarly identified the genes that have a decreasing pattern of expression with cancer progression.

**Figure 4.**
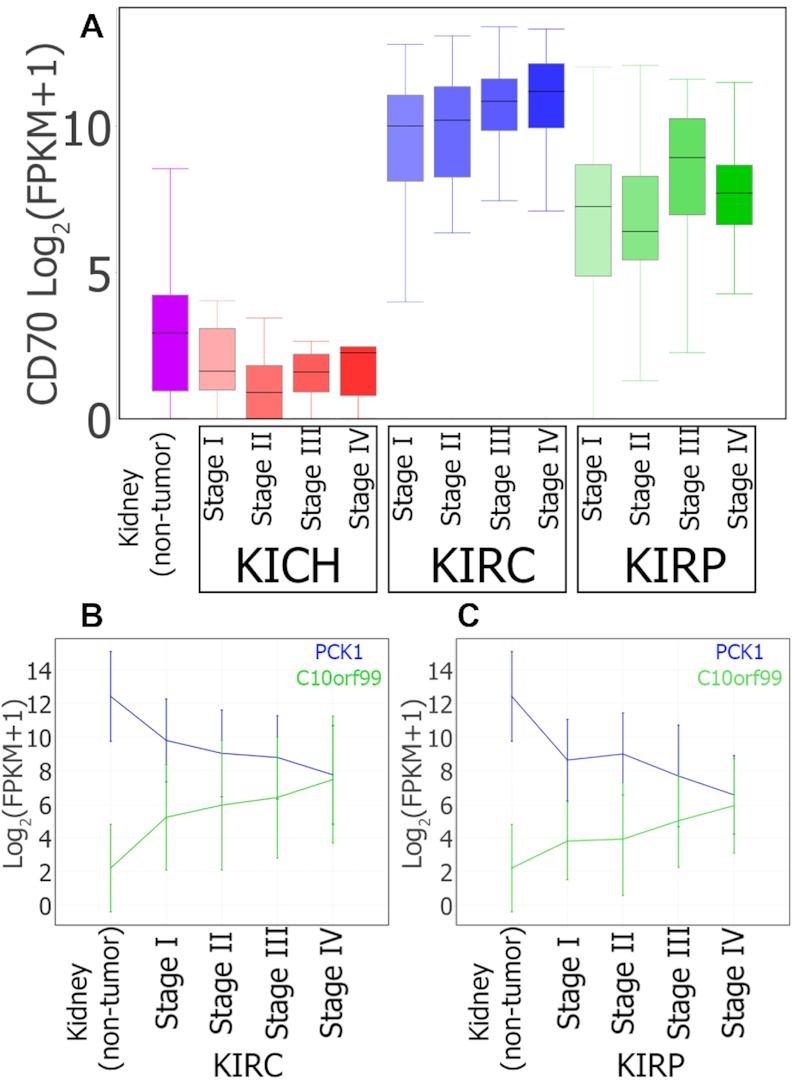
MOG visualization of expression of selected genes during progression of three types of renal cancer. (**A**) Box plots summarizing CD70 expression in non-tumor kidney and in different stages of KICH, KIRC and KIRP cancer progression. CD70 is designated as prognostic unfavorable for renal cancer by THPA (80). However, although CD70 levels in tumor samples increase 93-fold in KIRC and 14-fold in KIRP, CD70 levels *decrease* in KICH by 3-fold (*logFC* = −1.56; B-H corrected *P*-value = 0.004). (**B** and **C**) Line charts showing average expression of PCK1 (blue) and C10orf99 (green) over different stages of KIRC (B) and KIRP (C). The vertical lines are error bars. THPA designates PCK1 as prognostic favorable and C10orf99 as prognostic unfavorable for renal cancer.

ESCA, KIRP, KIRC THCA all included metadata and had sufficient numbers per stage to detect differentally expressed genes. (Full results in Additional file 4.) MOG reveals 221 genes that increase expression during tumor progression (gene numbers for each tumor type are: ESCA:91, KIRP:89, THCA:25, KIRC:24), and 227 genes that decrease expression (gene numbers for each tumor type: ESCA:89, KIRP:68, LIHC:64, KIRC:13) (Supplementary File 6 and Additional File 4). Of these 448 genes, 122 are flagged as prognostic markers by The Human Protein Atlas (THPA), which identifies prognostic markers by survival analysis (80). For example, Figure 4B and C shows the expression pattern of two such genes, Phosphoenolpyruvate Carboxykinase 1 (PCK1, known to be downregulated in KIRC (81) and general marker of renal failure (82)) and Chromosome 10 Open Reading Frame 99 (C10orf99, a known colon cancer inhibitor (83), and positive marker of KIRC (84)), in KIRC and KIRP.

Three hundred and twenty-seven genes that were identified in our study as differentially expressed in at least one tumor type were *not* labeled as prognostic in THPA (Supplementary File 6). For example, out of the 111 genes that increase during progression of KIRC or KIRP, only 56 were flagged as unfavorable prognostic for renal cancer by THPA. Of the 79 genes MOG identifies as decreasing with cancer progression in KIRC or KIRP, 39 were labeled as prognostic favorable for renal cancer by THPA. Twenty-seven genes out of the 64 that we identified by MOG as decreasing with cancer progression in LIHC were labeled by THPA as prognostic favorable for liver cancer. Out of 25 genes identified as having increasing pattern in THCA, none were labeled as prognostic by THPA. We propose that these genes may provide new candidates as biomarkers for prognosis of these tumor types (Supplementary File 6).

A number of the 327 genes identified as differentially expressed in MOG but not listed in THPA have been experimentally evaluated for their potential as prognostic markers (Table 3). For example, ARG1, CYP2C8, CYP3A4, CYP3A7 and CYP4A11, which we identified using MOG as decreasing expression with LIHC progression, have each been recently studied as prognostic markers for hepatocellular carcinoma (85–88). MOG analysis provides additional support for use of these genes as biomarkers.

**Table 3.** Genes identified by MOG as showing changing expression with cancer progression (B-H corrected *P*-value < 0.05) that had been identified in experimental studies as potential prognostic biomarkers but were not marked as prognostic for the given cancer type in The Human Protein Atlas (THPA) (80)

Disease	Gene	Gene name	Pattern	Ref.
LIHC	ARG1	arginase 1	Decreasing	(85)
LIHC	CYP2C8	cytochrome P450	Decreasing	(86)
		family 2 subfamily		
		C member 8		
LIHC	CYP3A4	cytochrome P450	Decreasing	(87)
		family 3 subfamily		
		A member 4		
LIHC	CYP3A7	cytochrome P450	Decreasing	(87)
		family 3 subfamily		
		A member 7		
LIHC	CYP4A11	cytochrome P450	Decreasing	(88)
		family 4 subfamily		
		A member 11		
THCA	CHI3L1	chitinase 3 like 1	Increasing	(119)
THCA	SFTPB	surfactant protein B	Increasing	(120)
THCA	CD207	CD207 molecule	Increasing	(121)
THCA	MUC21	mucin 21, cell	Increasing	(121)
		surface associated		
THCA	MMP7	matrix	Increasing	(122,123)
		metallopeptidase 7		
THCA	IGFL2	IGF like family	Increasing	(121)
		member 2		
THCA	KLK7	kallikrein related	Increasing	(124,125)
		peptidase 7		
THCA	FN1	fibronectin 1	Increasing	(126)

Using MOG to analyze and visualize the results by tumor type can reveal more nuanced information. For example, the Cluster of Differentiation 70 (CD70) gene is flagged by THPA and high CD70 expression is prognostic unfavorable for renal cancer. MOG analysis shows CD70 expression is higher in two types of renal tumors, KIRC and KIRP, and increases with disease progression (Figure 4A), but CD70 levels in another renal tumor type, KICH, have slightly *lower* expression than in non-tumor samples; thus, specifically in the case of KICH, *low* CD70 levels might be an unfavorable prognosis.

For prognosis and personalized medicine (89,90) *exceptions* can be extremely important, because specific tumor sub-types might respond differently to a particular treatment. By using MOG to explore RNA-Seq from large numbers of conditions and organs, a researcher can visualize data for individual samples or groups of that show changed expression of a prognostic marker or sets of markers, and compare these to those that do not.

Such exploration could suggest statistical analyses to try out in other, independent datasets to determine whether subsets of non-canonical samples might have a biologically distinct signature, revealing a different modality for a particular cancer. This in turn could be followed up by targeted experimental approaches or clinical studies.

### Exploring genes of unknown functions in *AT-microarray-dataset*

Our aim in the case study of *AT-microarray-dataset* was to explore patterns of expression of genes with little or nothing known about them. The well-vetted dataset we used (23), encompasses expression values for 22 746 genes across 424 *A. thaliana* samples, representing 71 diverse studies and a wide variety of environmental, genetic and developmental conditions (23). We updated the gene metadata to the current TAIR annotations (33) and added phylostrata designations (obtained from phylostratr (59)).

**Figure 5.**
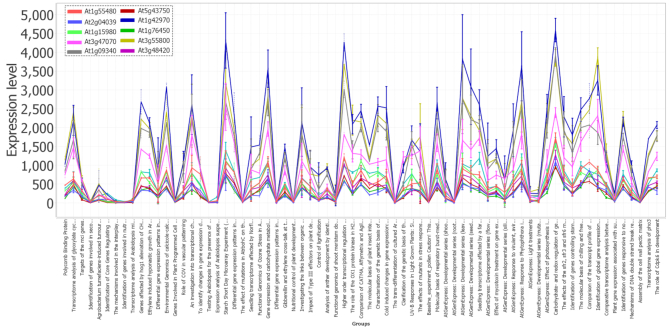
Spearman correlation followed by line-plot visualization, using MOG, shows that Met1 (At1G55480) is highly expressed in photosynthetic organs and highly correlated with several genes of unknown function. The ‘peaks’ of expression are all leaf samples; the ‘troughs’ of expression are predominantly root and cell culture samples. *AT-microarray-dataset* representing 71 diverse studies and a wide variety of environmental, genetic and developmental conditions (23). Several genes of unknown function are closely co-expressed in this cluster.

We sought to identify genes of unknown or partially known function that might be involved in photosynthesis, the process that gave rise to the earth’s oxygenated atmosphere and the associated evolution of extant complex eukaryotic species. We focused particularly on regulation of the assembly and disassembly of the photosystem I and II light harvesting complexes; these dynamic processes respond sensitively to shifts in light and other environmental factors (91–94). In particular, Met1 (AT1G55480) is a 36 Kda protein that regulates the assembly of the photosystem II (PSII) complex (94). To explore genes that might be involved in PSII assembly, we calculated Spearman correlation of Met1 expression with that of the 22 746 genes represented on the Affymetrix chip (Figure 5). This analysis finds 104 genes whose expression is highly correlated to Met1 (Spearman’s coefficient > 0.9) across all conditions (Supplementary File 7).

We examined whether genes of photosynthesis were over-represented in this Met1-co-expressed cohort. Among the Met1 co-expressed genes, the Gene Ontology (GO) Biological Functional terms most highly over-represented (*P*-value < 10^−5^) are integral to the light reactions of photosynthesis: generation of precursor metabolites and energy; photosynthetic electron transport in photosystem I (PSI); reductive pentose-phosphate cycle; response to cytokinin; and PS2 assembly (Supplementary File 7). For example, the gene most highly correlated with Met1 is At2g04039, a gene encoding the NdhV protein, which is thought to stabilize the nicotinamide dehydrogenase (NDH) complex of PS1 (95); phylostratal analysis (59) indicates that NdhV has homologs across the photosynthetic organisms, streptophyta (land plants and most green algae). Eighteen of the Met1 co-expressed genes are designated as ‘unknown function’ or ‘uncharacterized’; six are restricted to Viridiplantae. These genes would be good candidates to evaluate experimentally for a possible function in photosynthetic light reaction.

**Figure 6.**
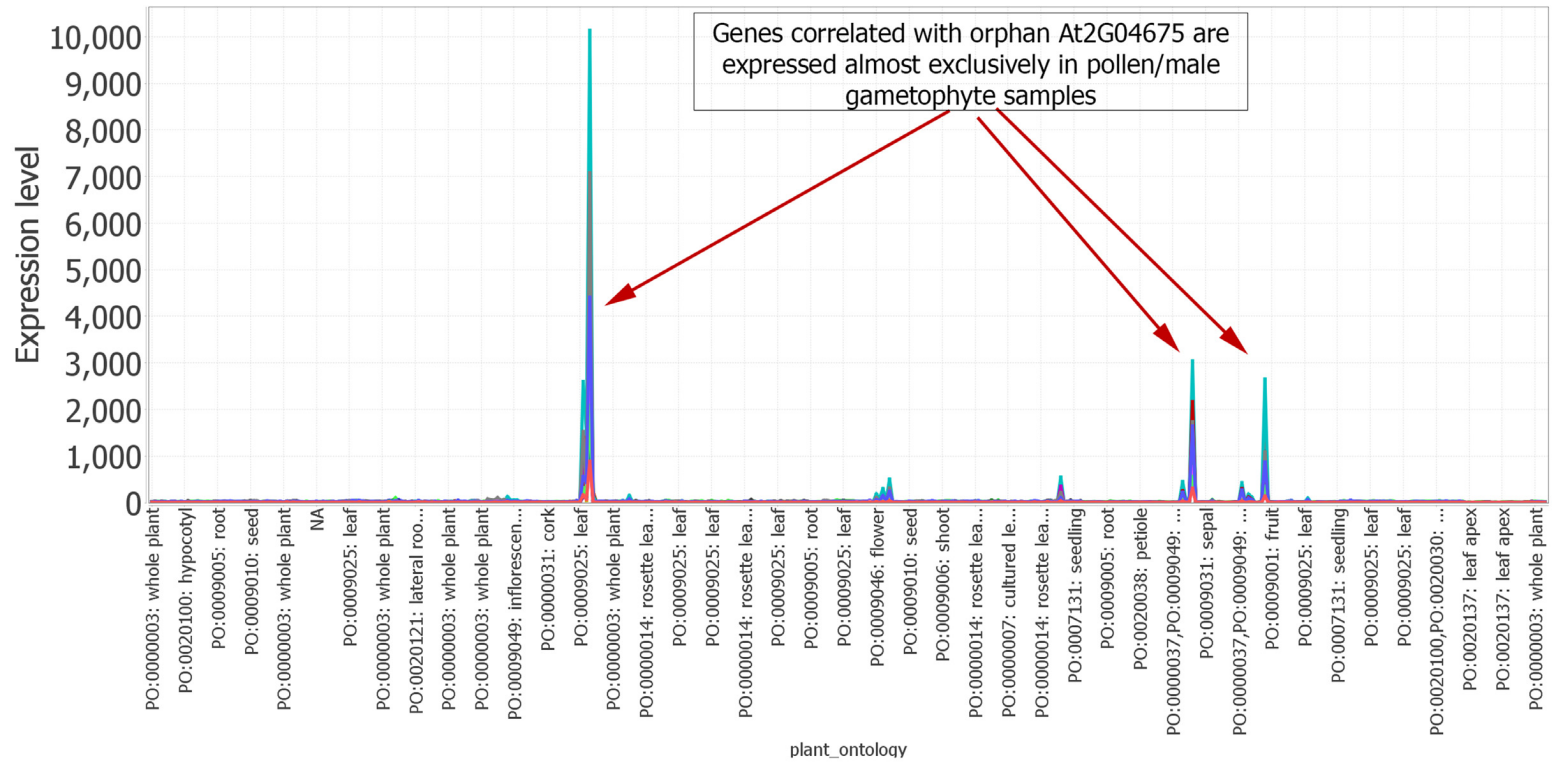
MOG line chart visualization shows the expression of orphan gene At2G04675 over the *AT-microarray-dataset* representing 71 diverse studies and a wide variety of environmental, genetic and developmental conditions (23). X-axis are samples, and Y-axis indicates their expression value. The orphan gene At2G04675 is of no known function, and genes highly correlated with At2G04675 are expressed almost exclusively in pollen/male gametophyte samples. Each line represents a gene. (Lines in this visualization are for clarity and the connections from sample to sample do not imply a relationship).

Our next aim was to use MOG to directly explore an orphan gene (a gene encoding a protein unrecognizable by homology to those of other species) (59,96–97), and to determine potential processes that it might be involved in. First, we filtered each gene’s target description to retain ‘unknown’. From these, we filtered to retain only the phylostratigraphic designation ‘*A. thaliana*’. From this gene list, we identified genes that had an expression value greater than 100 in at least five samples. We selected the orphan gene of unknown function, At2G04675, for exploratory analysis. At2G04675 encodes a predicted protein of 67 aa with no known sequence domains (domains searched using CDD (98)). A Pearson correlation analysis of the expression pattern of At2G04675 with the other genes represented on the Affymetrix chip showed 48 genes had a Pearson correlation of higher than 0.95 (Supplementary File 7); these genes are expressed almost exclusively in pollen (the male gametophyes of flowering plants) (Figure 6). The exploration implicates At2G04675 as a candidate for involvement in some aspect of pollen biology.

**Figure 7.**
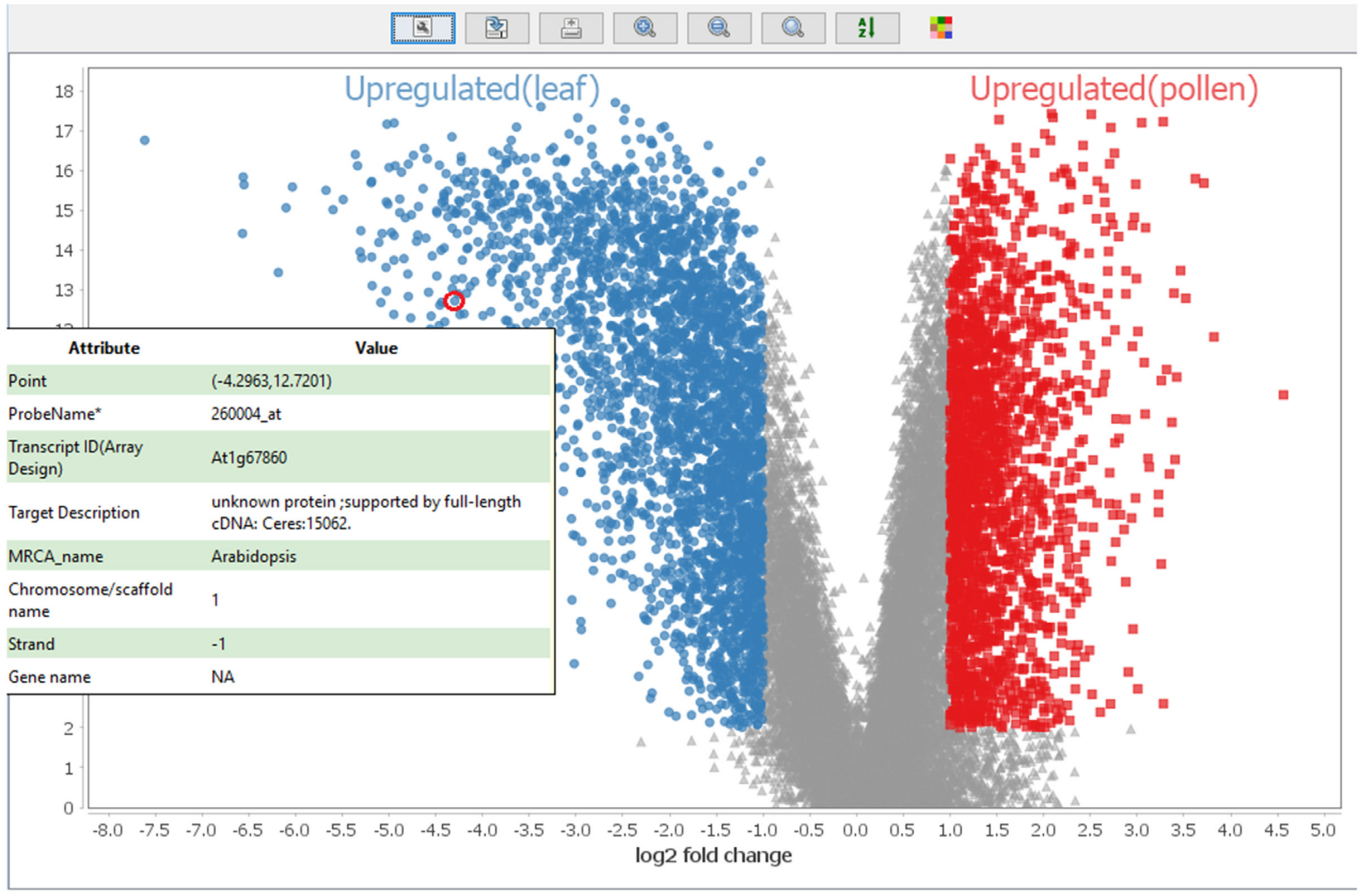
Using MOG for differential expression analysis of leaf and pollen samples, followed by volcano plot visualization (Y-axis: -log_10_(*P*-value)). At1G67860, an Arabidopsis specific gene with no known function, is 16-fold more highly accumulated in leaves relative to pollen (Mann–Whitney U test; BH corrected *P*-value<10^−3^). The gene metadata is revealed upon hovering the mouse over a data point.

Using MOG to further explore genes that are associated with pollen, we identified sets of leaf and pollen samples (Supplementary Figure S8 and Additional File 5), and then calculated genes that are differentially expressed in the leaf samples versus the pollen samples using a Mann–Whitney U test (fold change of 2-fold or more; BH corrected *P*-value < 10^−3^) (Additional File 5). The GO terms most highly enriched (*P*-value < 10^−20^) among genes upregulated in pollen are processes of cell cycle, mitosis, organellar fission, chromosome organization and DNA repair (Additional File 5). This reflects and emphasizes the critical role of these processes in male gametophyte development, particularly sperm biogenesis. Each angiosperm pollen grain must produce two viable sperm each used in the double fertilization of the ovule. Above all else, proper mitogenesis is essential to the function of a pollen grain. We visualized the *leaf versus pollen* differential analysis by volcano plot (Figure 7 and Supplementary Figure S9), this time to explore genes upregulated in leaf. Among these is At1G67860, an Arabidopsis specific gene encoding a protein of ‘unknown function’. We used MOG to correlate expression of this gene versus all genes across all samples. One hundred sixteen genes, dispersed across all five chromosomes, are co-expressed with At1G67860 (Spearman correlation ≥ 0.65) (Supplementary File 7). The genes are expressed almost exclusively in mature leaf (Supplementary Figure S10). Most have no known function; a GO enrichment test indicates that GO biological processes overrepresented (*P*-value < 10^−3^) among the genes are: defense response, response to stress, response to external biotic stimulus and response to other organism (Supplementary File 7).

### Identifying co-expressed metabolites in *AT-metab-dataset*

Metabolomics is providing a growing resource for understanding metabolic pathways and identifying the structural and regulatory genes that shape these pathways and their interconnected lattice (61,99–100). Here, we use the *AT-metab-dataset* metabolomics dataset that represents a comprehensive study of 50 mutants with a normal morphological phenotype but altered metabolite levels, and 19 wild-type control lines (60). There are 8–16 biological replicates for each genetic line; data is corrected for batch effects. Data and metadata were retrieved from PMR (61). The aim of this case study was to tease out co-expressed metabolites that are affected by genetic perturbations. We identified a group of four highly co-expressed metabolites (Pearson correlation > 0.8): the amino acid arginine, its precursor L-ornithine, cyclic ornithine (3-amino-piperidine-2-one), and one unidentified metabolite. Plots across the means of the biological replicates of each sample (Supplementary Figure S11), shows accumulation of these metabolites is upregulated over 4-fold in four mutant lines: *mur9*, mutants have altered cell wall constituents; *vtc1*, encodes GDP-mannose pyrophosphorylase, required for synthesis of manose, major constituent of cell walls, upregulated upon bacterial infection; *cim13*, gene of unknown function associated with disease resistance, *eto1*, negative regulator of biosynthesis of the plant hormone ethylene. An arginine-derived metabolite, nitrous oxide, has been widely implicated in signaling pathways in plants (101). MOG analysis might suggest to a researcher a potential relationship between arginine and the cell wall defense response, providing a suggestion for future experimentation.

### Comparison to other software

Few tools that do not require coding are available for on-the-fly exploration of expression data. Most are ‘shiny’ (13) apps (15–18,102) providing a web interface to a limited number of R packages for data visualization, batch correction, differential expression analysis, PCA analysis (among samples) and gene enrichment analysis. Although shiny (13) is constantly improving, existing tools written in R (15–17) must rely on R’s present capabilities for interactive applications (103). In contrast to R, Java, MOG’s platform, has been used to develop numerous software with interfaces that are interactive and user-friendly (e.g. (78,104–106)), and MOG provides the researcher with specialized GUIs and methods for exploratory data analysis. MOG’s GUI allows direct interactivity with the data through interactive tables, trees and visualizations, so that a researcher can easily explore data from different perspectives.

Most available R-based tools read all data directly into the main memory. Thus, on a laptop/desktop computer, analysis of a big dataset is slow (or crashes) if the available memory is not sufficiently large. For example, a dataset of 100 000 human transcripts over 5000 samples (500 000 000 expression values) requires at least 4GB (8 byte for each value) of free memory to be loaded into memory at once. To circumvent this problem, R developers can use the new DelayedArray (107) framework together with DelayedMatrixStats (108) which can enable efficient handling of big datasets with R. For example, iSEE’s (18) code is compatible with using DelayedArray (107) objects.

In contrast, MOG uses an indexing strategy to read data only when it is needed, which drastically reduces the total memory consumption of the system. Table 4 compares five of the most recent tools for exploratory analysis of expression data to MOG. (More details are provided in Supplementary File 8.)

**Table 4.** MOG compared to existing tools for exploratory analysis of expression data

	MOG	PIVOT	iSEE	iGEAK	IRIS-EDA	DEvis
Reference	This paper	(15)	(18)	(16)	(17)	(102)
Year	2019	2018	2018	2019	2019	2019
Platform/GUI	Java/Swing	R/Shiny	R/Shiny	R/Shiny	R/Shiny	R/None
Interactive tables and trees	Yes	No	No	No	No	No
Interactive drag and drop operations	Yes	No	No	No	No	No
Interactive visualizations	Yes	Partial	Partial	Partial	Partial	No
Interactively subset data	Yes	Partial	Partial	Partial	No	No
Save progress	Yes	Yes	Partial (if user saves R code)	No	No	No
Use any R package	Yes	No	No	No	No	No
Supported data types	Omics or other numerical data	RNA-Seq/scRNA-Seq	Omics	RNA-Seq/microarray	RNA-Seq/scRNA-Seq	RNA-Seq
MW U test (sec.)	7	1260	NA	NA	NA	NA

MOG’s GUI, designed with Java swing, is fully interactive; in contrast, other available tools are based on R and provide limited or no interactivity. A MOG user can execute any R package/script with interactively selected subsets of data if s/he wishes to perform additional analysis, whereas only a limited number of R-packages are available in the other tools. The last row compares the Mann–Whitney U test’s execution time for MOG and PIVOT using the liver tumor and non-tumor datasets (18 212 genes over 410 samples). A more detailed comparison of the tools is available in Supplementary File 8.

#### Benchmarking

We benchmarked MOG’s performance with the *Hu-cancer-RNASeq-dataset* (18 212 genes over 7142 samples) using a laptop with 64 bit Windows 10, 8 GB RAM and Intel(R) Core(TM) i5-7300HQ CPU; the system’s resource utilization was monitored by Windows Performance Monitor tool (WPMT) (109). During benchmarking, only the software being tested was running. MOG’s efficiency was compared to that of one of the R-based ‘shiny’ web-app (13) (choosing PIVOT, because it permits loading normalized data).

**Figure 8.**
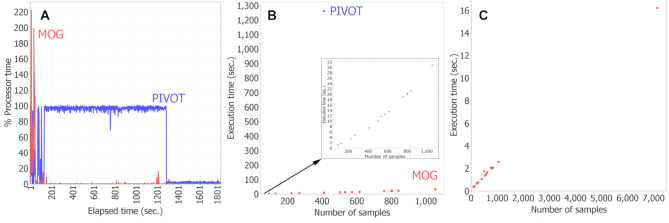
MOG performance benchmarks. MOG was benchmarked using the entire *Hu-cancer-RNASeq-dataset* (18 212 genes over 7142 samples), and using chunks of this dataset.(**A**) Comparison of MOG to R-based (PIVOT). Dataset size was limited to the amount of data that could be loaded in PIVOT (410 samples). % processor time (% CPU utilization) was calculated over 30 min; theoretical maximum value = total processors in computer x 100 (400 in this case). (**B**) Execution times for computing differentially expressed genes using Mann–Whitney U test. Red dots, MOG; blue dot, PIVOT (410 samples). Inset, expanded scale to display MOG execution times. (**C**) MOG execution times for pairwise computations of Pearson correlation of a gene (BIRC5) with all other genes in the datasets. (Other tools cannot perform this computation). Execution times are linear with data size; full dataset analysis took 16 s.

PIVOT repeatedly crashed and failed to load the full *Hu-cancer-RNASeq-dataset* (Additional File 6), but was able to load a subset of data consisting only of 410 tumor and non-tumor liver samples. We measured the execution time (time taken to compute and display output) of the Mann–Whitney U test for differentially expressed genes in tumor versus non-tumor samples. The test completed in 21 min with PIVOT, but only seven seconds with MOG (Figure 8). We kept MOG running idle until total runtime reached 30 min and compared memory and processor usage (Supplementary File 8); average memory usage of PIVOT (1869 MB) was about twice that of MOG (995 MB) (Supplementary File 8). Peak % processor time (CPU) was greater for MOG; however, MOG completed its task much more quickly, and over the 30 min, the *average* % processor time was 64% for PIVOT but only 2% for MOG (Figure 8A).

We benchmarked MOG’s performance on datasets of different sizes, created by splitting the *Hu-cancer-RNASeq-dataset* by organ type (tumor and non-tumor samples). For each dataset, we performed a Mann–Whitney U test on all the genes for tumor versus non-tumor groups. MOG took only 31 s to compute a Mann–Whitney U test on 18 212 genes over 1054 samples (Figure 8B and Additional File 6). We then measured the execution time for calculating Pearson correlations of one gene with all others. MOG took only a couple of seconds to compute a Pearson correlation over 1000 samples and 16 s to compute over 7142 samples (Figure 8C).

## DISCUSSION AND CONCLUSION

We demonstrated MOG’s functionalities by exploring three different well-validated datasets: a human RNA-Seq dataset from non-tumor and tumor samples (*Hu-cancer-RNASeq-dataset*), an *A. thaliana* microarray dataset (*AT-microarray-dataset*) and an *A. thaliana* metabolomics dataset (*AT-metab-dataset*). In each case, known information was recapitulated in the MOG analysis, and new potential relationships became apparent.

During exploration of the *Hu-cancer-RNASeq-dataset* by MOG, we created a catalog of genes that are differentially expressed in different types of tumors, identifying in this process 35 genes that are consistently upregulated or downregulated in every type of cancer in the dataset. GPC3 (67,71–72) was identified by MOG as a biomarker gene for liver cancer. Gene-level resolution analysis by MOG revealed that the cadre of genes that are co-expressed with the GPC3 gene change drastically among the individual organs, and between tumor samples and corresponding non-tumor samples. By mining the sample and study metadata, we identified genes that showed regulation with cancer progression. Many of the genes we identified have been reported previously in the literature and in THPA to be prognostic biomarkers for different cancers. Many other genes that MOG identified as differentially expressed genes are *not marked as prognostic in THPA*. These genes present potential new biomarkers for disease progression. Because each tumor type has many variations, investigating multiple candidate prognostic markers in individual tumors can provide critical information for personalized medicine (110).

Using the *AT-microarray-dataset*, we explored expression patterns of genes with unknown functions including orphan genes, identifying 18 mostly plant-restricted genes that are tightly co-expressed with genes central to photosystem assembly. We also identified an Arabidopsis-specific gene, At2G04675, to be highly expressed in pollen development, suggesting a potential involvement of this gene in gametogenesis. With the *AT-metab-dataset*, we identified a potential relationship between arginine and the cell wall defense response. Such exploratory analyses provide clues as to how to approach experimentally testing the function of these genes or metabolites.

Processing multiple heterogeneous RNA-Seq data is a formidable and unsolved challenge. We have intentionally not added capabilities for data processing (e.g. alignment, normalization, and batch-correction to minimize unwanted technical and biological effects) into MOG for two reasons. First, the selection of appropriate statistical and computational methods depends on the data structure and the biological questions to be asked. Different types of data have different characteristics (111–113), and if statistical methods are misapplied during normalization and batch-correction, especially when the data are from multiple heterogeneous studies, the resultant dataset may be misleading. Much as if using R or MATLAB statistical software, a MOG user must consider these technicalities. Second, the data science field is far from unsettled (112–115) with new approaches and variations being developed each year. (GoogleScholar retrieved over 10 000 journal articles from the first half of 2019 for ‘RNA-Seq normalization methods’). Potentially a researcher could use MOG as a tool to compare the results of different methods of processing the same raw data. Such interactive comparisons would enable biologists to gain insight as to which processing methods best reflect experimentally-established ‘ground truths’. This approach would provide a complement to the more typical validation of a dataset by determining GO term enrichment in gene clusters.

Analyses performed while exploring and statistically analyzing datasets on MOG can be saved; by clicking ‘save’, all the analyses that have been performed are added as objects to the MOG project file. Results obtained with MOG can be shared by sharing the saved MOG project file. If a user wishes to document the information to reproduce the analysis, she/he needs to manually specify the parameters and methods used. In the future, we plan to implement automated report generation for each analysis.

MOG is a novel Java software for interactive exploratory analysis of big ’omics datasets or other datasets. By using an indexing strategy to read data only when it is needed, the total memory consumption of the system is minimized, enabling MOG to perform much more efficiently than the available R-based software. Visualizations produced by MOG are fully interactive, and enable researchers to detect and mine interesting data points and probe the relationships among them. The statistical methods implemented in MOG help to guide exploration of hidden patterns in a user-friendly manner. By integrating metadata, MOG affords an opportunity to extract new insights into the relationships between gene expression and gene structure, gene location, or any of the diverse information entered by scientists about the biology and experimental conditions.

Taken together these features can aid a researcher in developing new, experimentally testable hypotheses.

## DATA AVAILABILITY

We subscribe to FAIR data and software practices (116). MOG is free and open source software published under the MIT License. MOG software, user guide and all compiled datasets in this article are freely downloadable from http://metnetweb.gdcb.iastate.edu/MetNet_MetaOmGraph.htm. MOG’s source code and user guide is available at https://github.com/urmi-21/MetaOmGraph/. MOG’s source code (version 1.8.0) at the time of submission is archived and can be accessed using the DOI:10.5281/zenodo.3520986. Additional files are available at https://github.com/urmi-21/MetaOmGraph/tree/master/MOG_SupportingData.

## Supplementary Material

gkz1209_Supplemental_Files
