# Supplementary material for: MetaOmGraph: a workbench for interactive exploratory data analysis of large expression datasets
Source: Nucleic Acids Res. 2020 Jan 20;48(4):e23. doi: 10.1093/nar/gkz1209 (PMC7039010; doi:10.1093/nar/gkz1209)
Supplement: gkz1209_Supplemental_Files [file gkz1209_supplemental_files.zip › oct 29SupplementaryFigures_.pdf]

MetaOmGraph: a workbench for interactive exploratory data analysis of large expression datasets (Supplementary Materials)

Urminder Singh[1,2,3], **Manhoi Hur**[2], **Karin Dorman**[1,3,4], **and Eve Syrkin Wurtele**[1,2,3*]

[1]Bioinformatics and Computational Biology Program, Iowa State University, Ames, IA 50011, USA
[2]Center for Metabolic Biology, Iowa State University, Ames, IA 50011, USA
[3]Department of Genetics Development and Cell Biology, Iowa State University, Ames, IA 50011, USA
[4]Department of Statistics, Iowa State University, Ames, IA 50011, USA
[*]e-mail: mash@iastate.edu

Contents

1 Supplementary Figures 1
References 12

1 Supplementary Figures

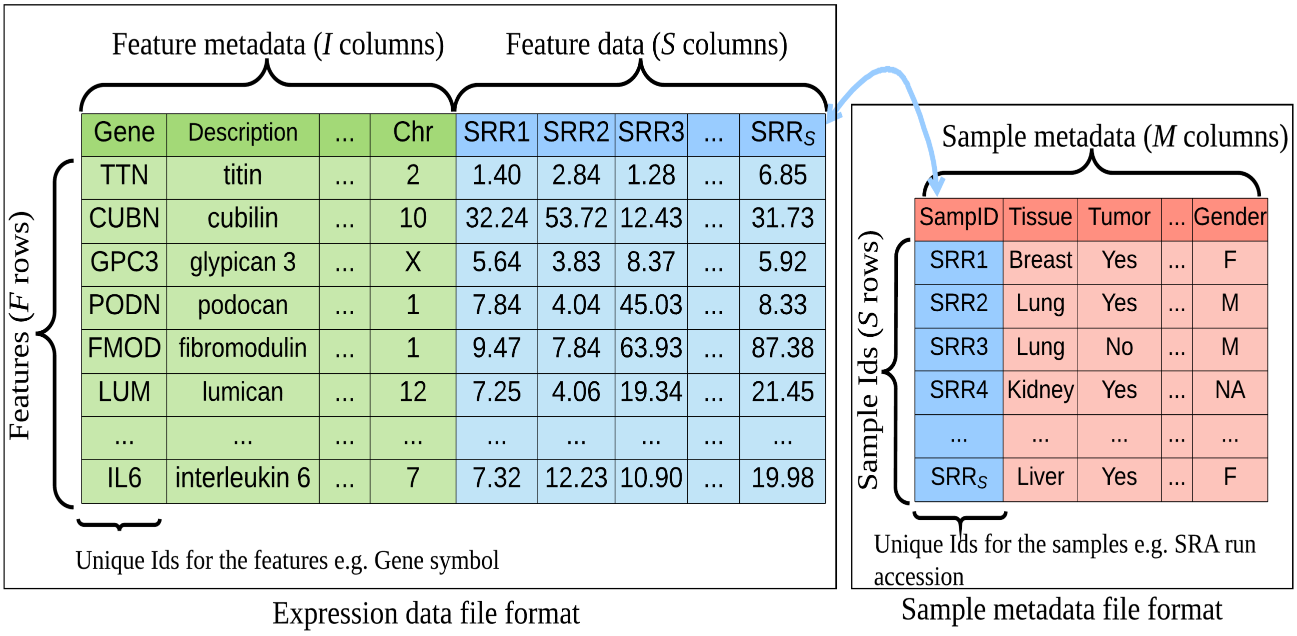

Supplementary Figure 1. Structure of input files for a new MOG project. The *expression data file* (left table) is a matrix with F rows and $(I+S)$ columns. Each row corresponds to a feature (in this example, the features are genes). The first I columns are feature metadata columns; the first column is a unique feature ID for each row (in this example, the gene name). The latter (S) columns are the expression values of F features over S samples. Each column is headed by a unique identifier for the sample. The *sample metadata file* (right table) is a matrix of S rows by M columns. Its first column contains the unique sample IDs. Thus, each row in the sample metadata file corresponds to a sample in the expression data file. The M columns are the metadata attributes of each analysis (e.g., each run for RNA-Seq data, each chip for microarray data).

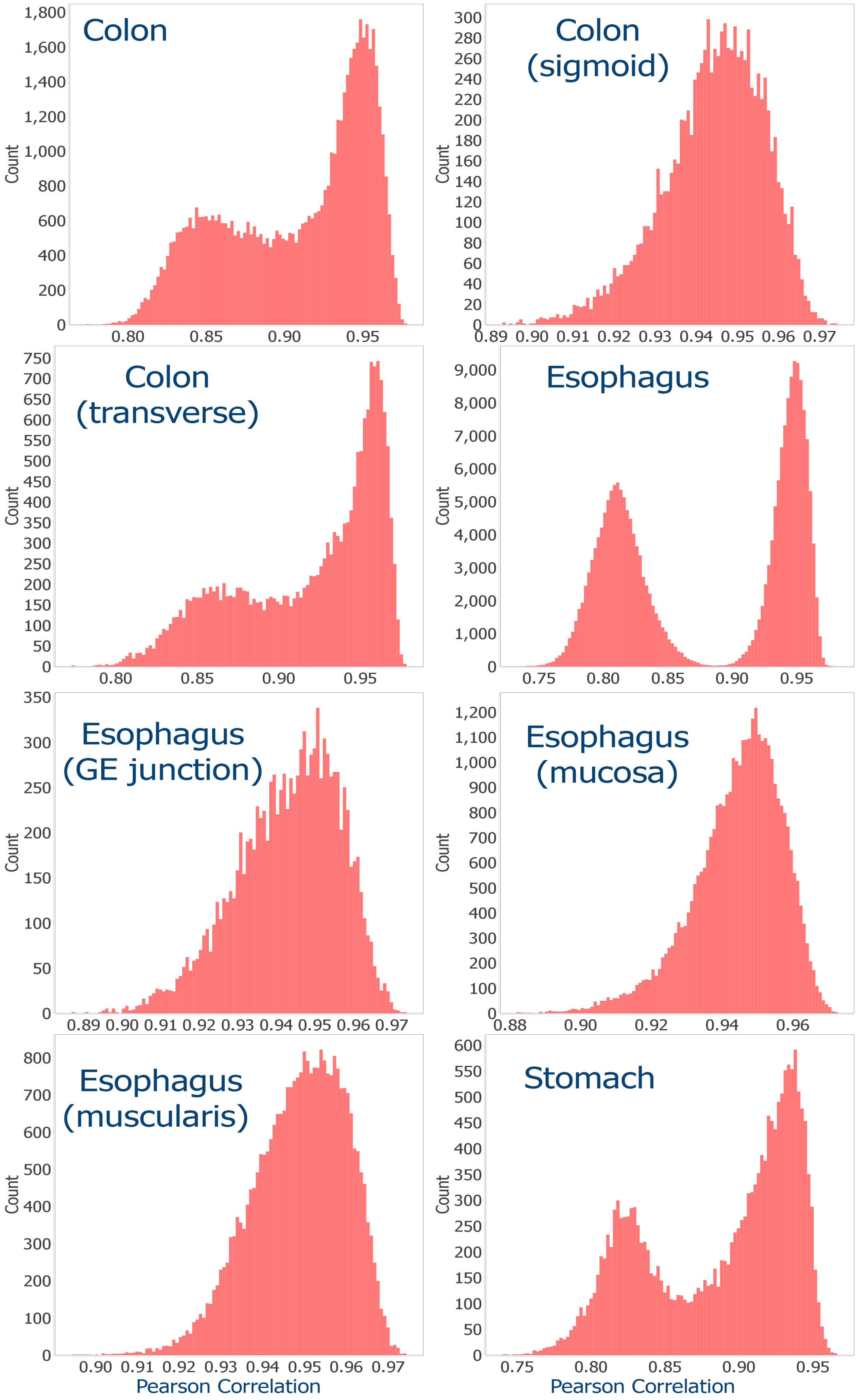

Supplementary Figure 2. The distribution of Pearson correlation values among samples from colon, esophagus and stomach tissues.

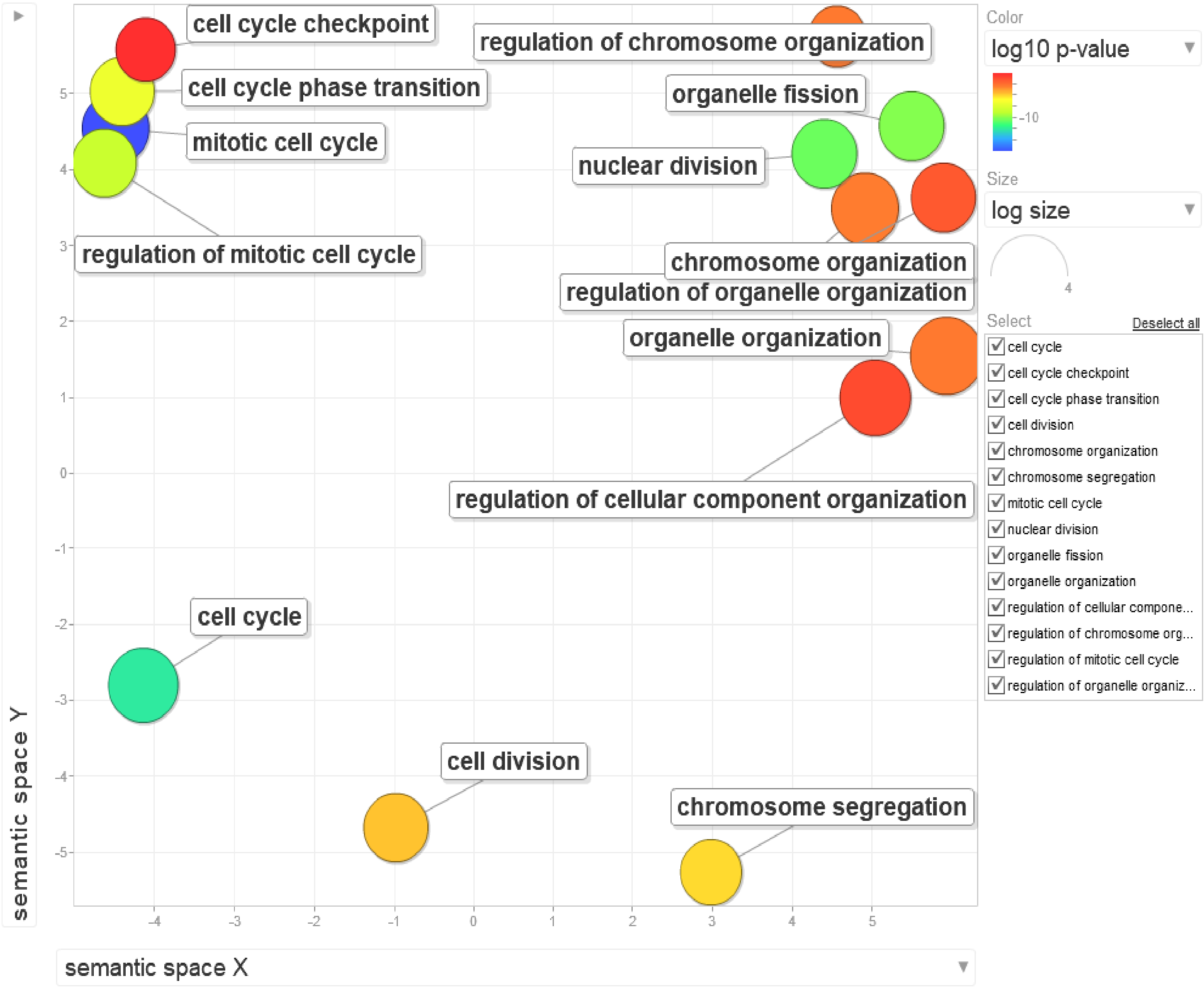

Supplementary Figure 3. GO terms enriched in the 16 upregulated genes in all 14 cancer types visualized using REVIGO[1].

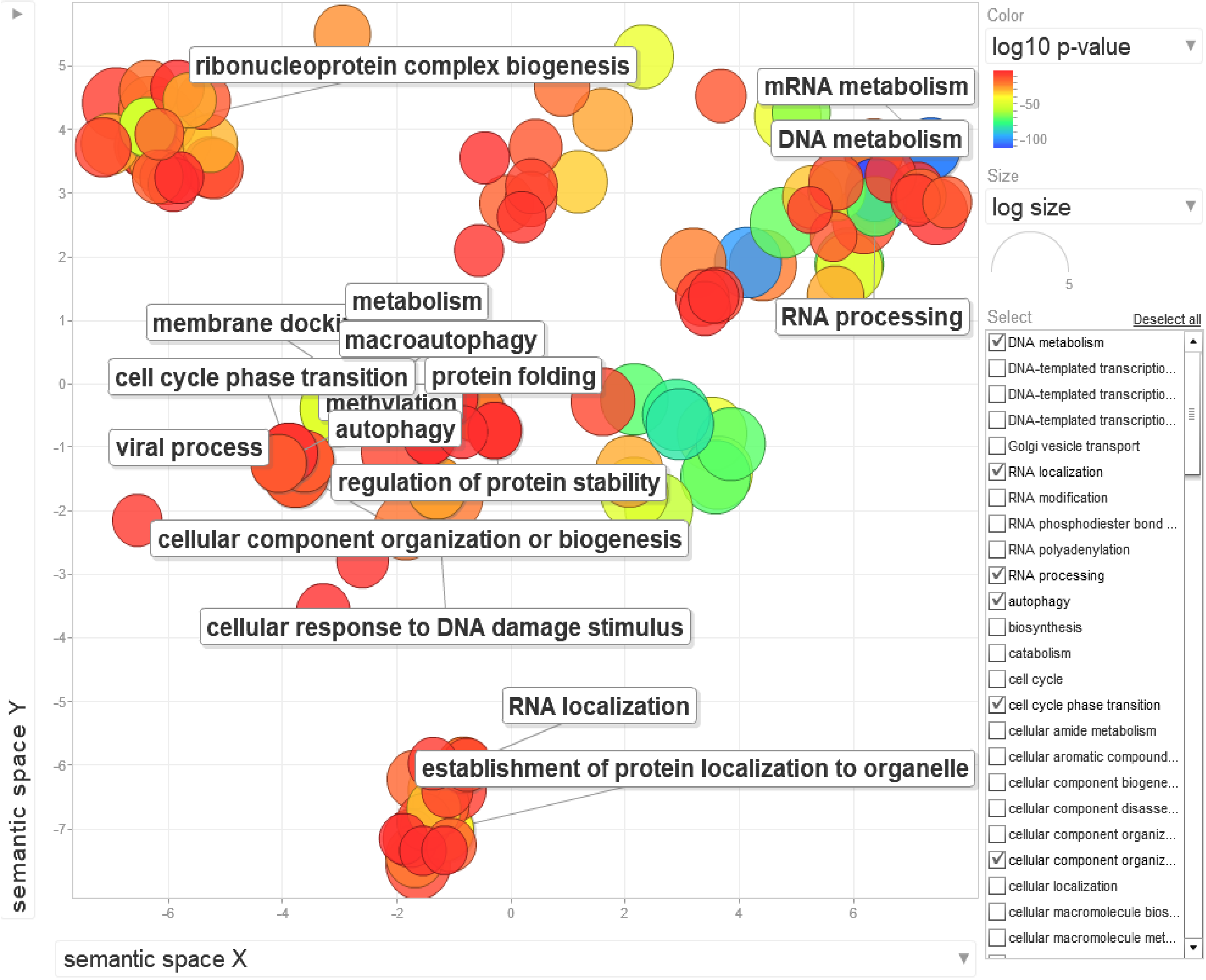

Supplementary Figure 4. GO terms enriched in the 5,784 unchanged genes in all 14 cancer types visualized using REVIGO[1].

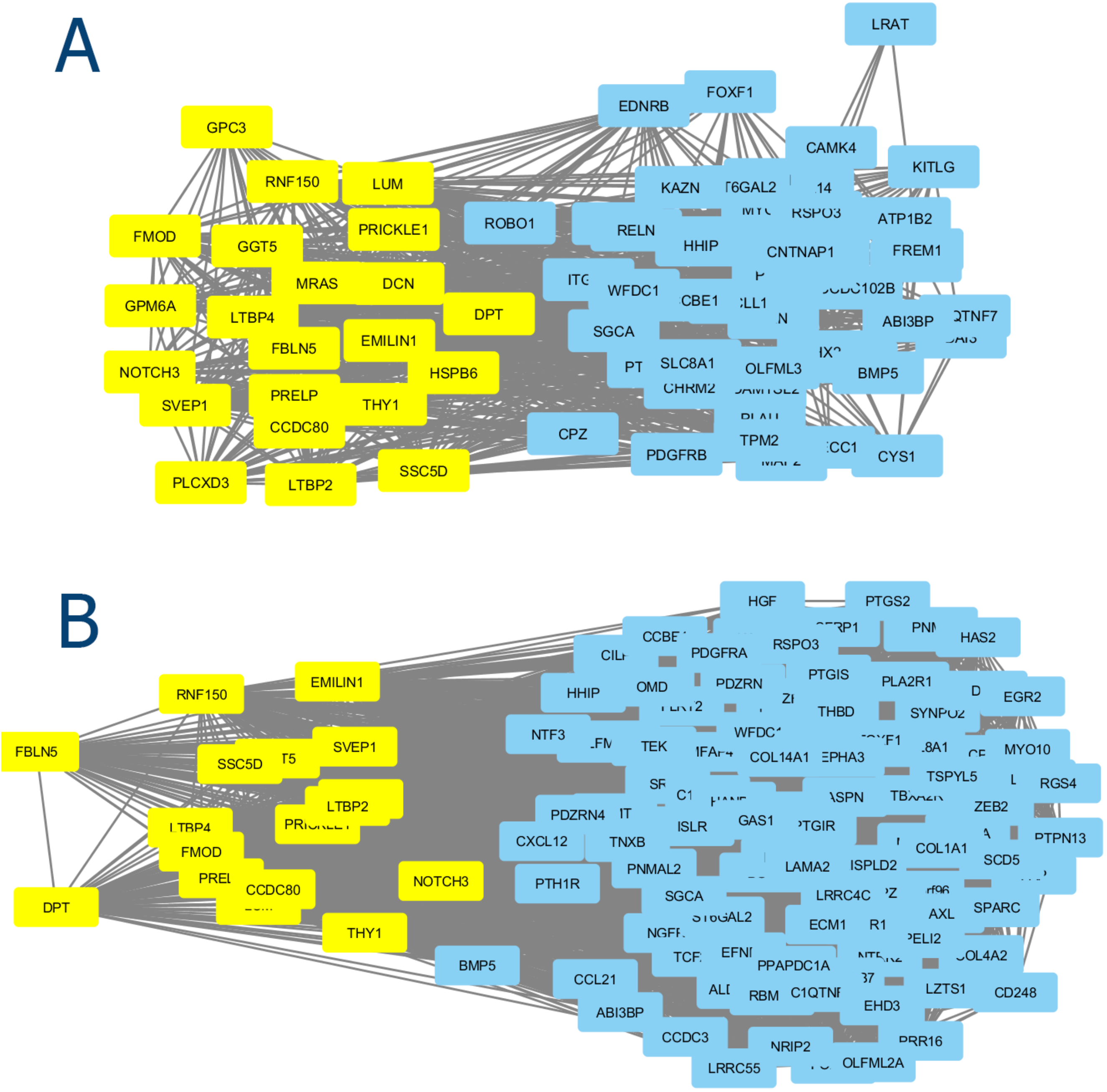

Supplementary Figure 5. Modules identified in coexpression networks made using 3,012 differentially expressed genes in LIHC vs normal liver samples and visualized using cytoscape[2]. (**A**) Shows the GPC3 containing module identified in the network from liver samples. GPC3 is directly connected to 21 genes (shown in yellow). (**B**) Shows the module found in the network inferred from LIHC samples. In this network GPC3 was absent, but this module shared 33 genes with that of from the liver normal network. Genes which were directly connected to GPC3 in first module are shown in yellow.

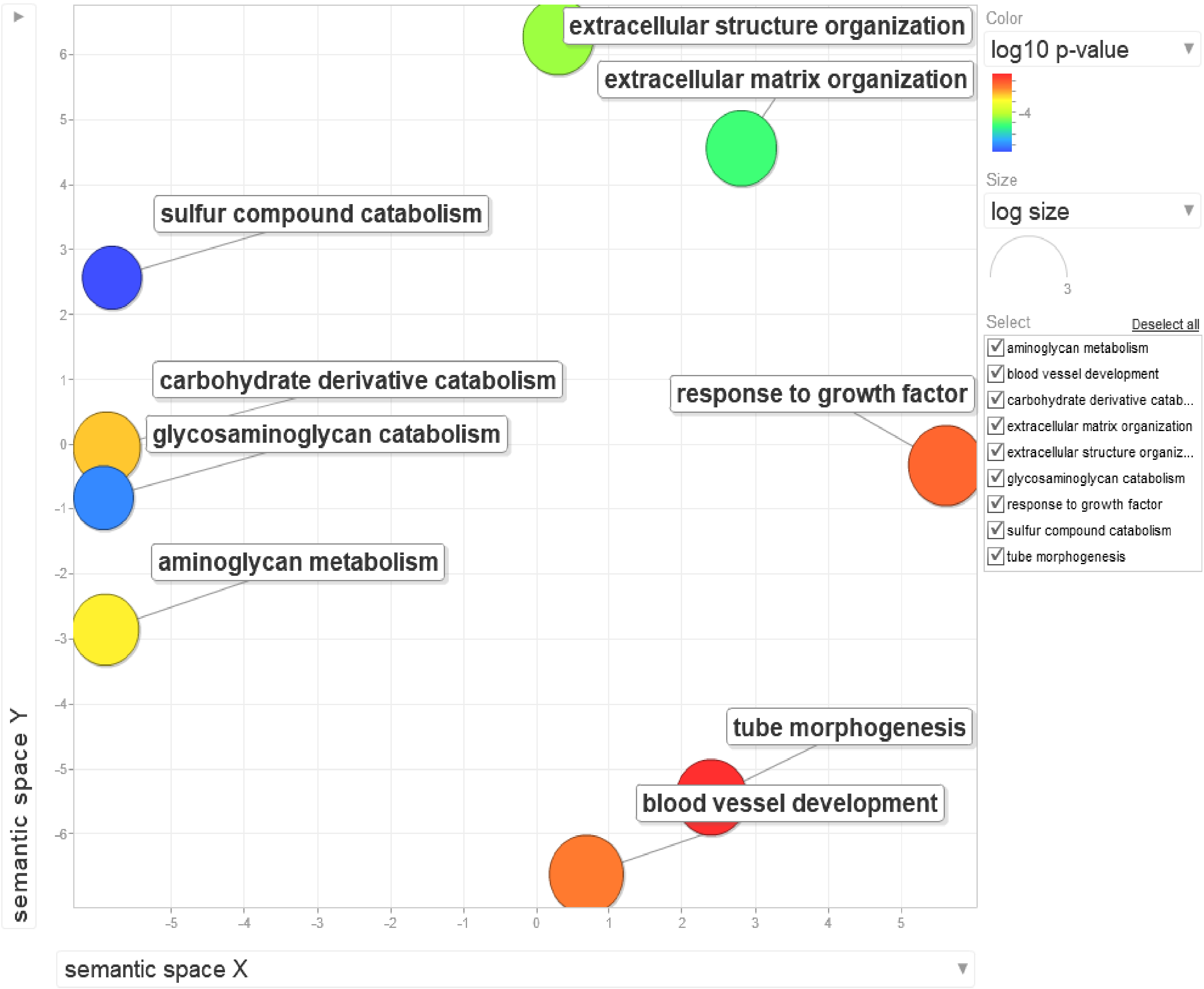

Supplementary Figure 6. GO terms enriched in the GPC3 containing module (Supplementary Figure 5 A) found in the coexpression network from the liver samples visualized using REVIGO[1].

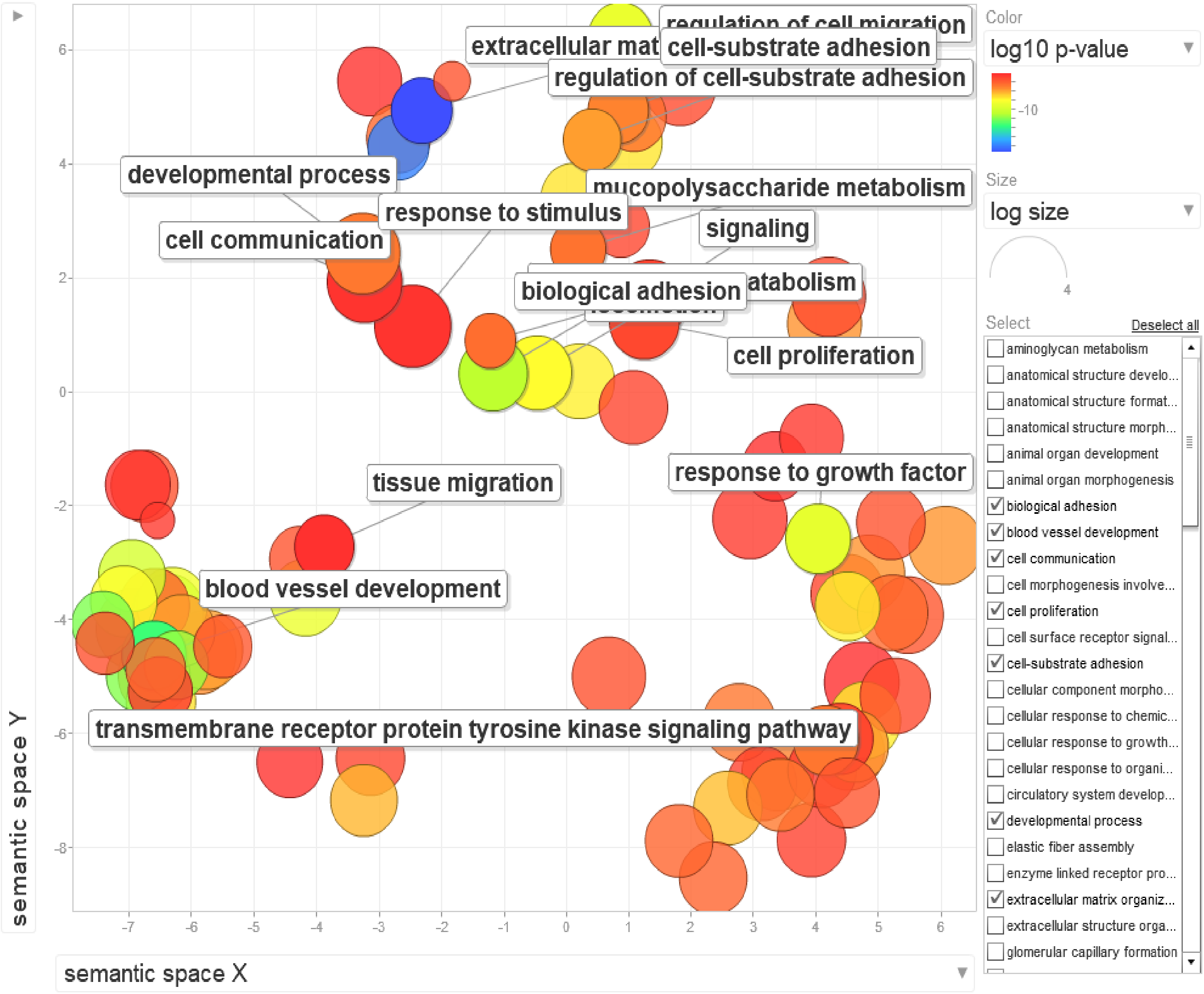

Supplementary Figure 7. GO terms enriched in the second module (Supplementary Figure 5 B) found in the coexpression network from the LIHC samples visualized using REVIGO[1].

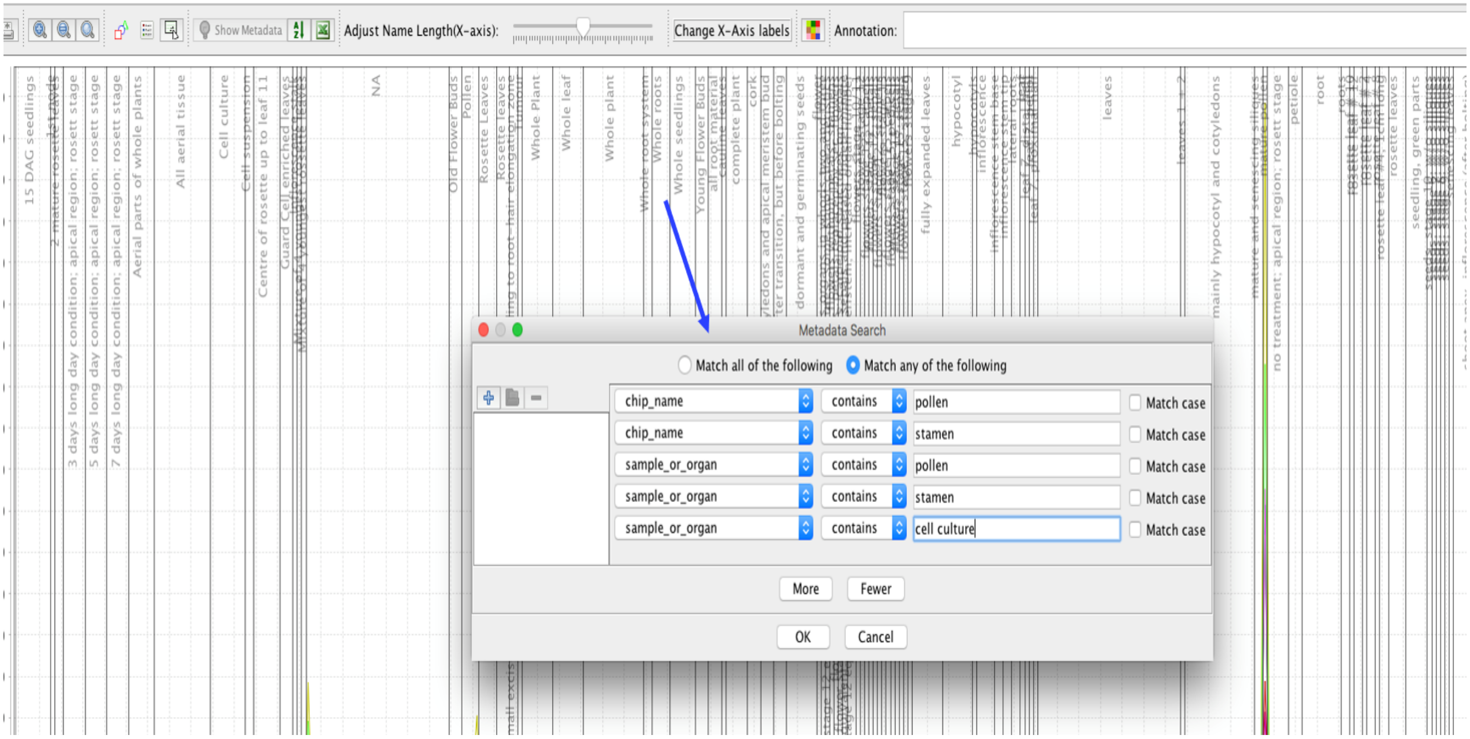

Supplementary Figure 8. Differential analysis of genes upregulated in pollen versus leaf- sample selection. Pollen samples were selected as in the inset. Leaf samples were selected by "leaf" in organs and plant ontology.

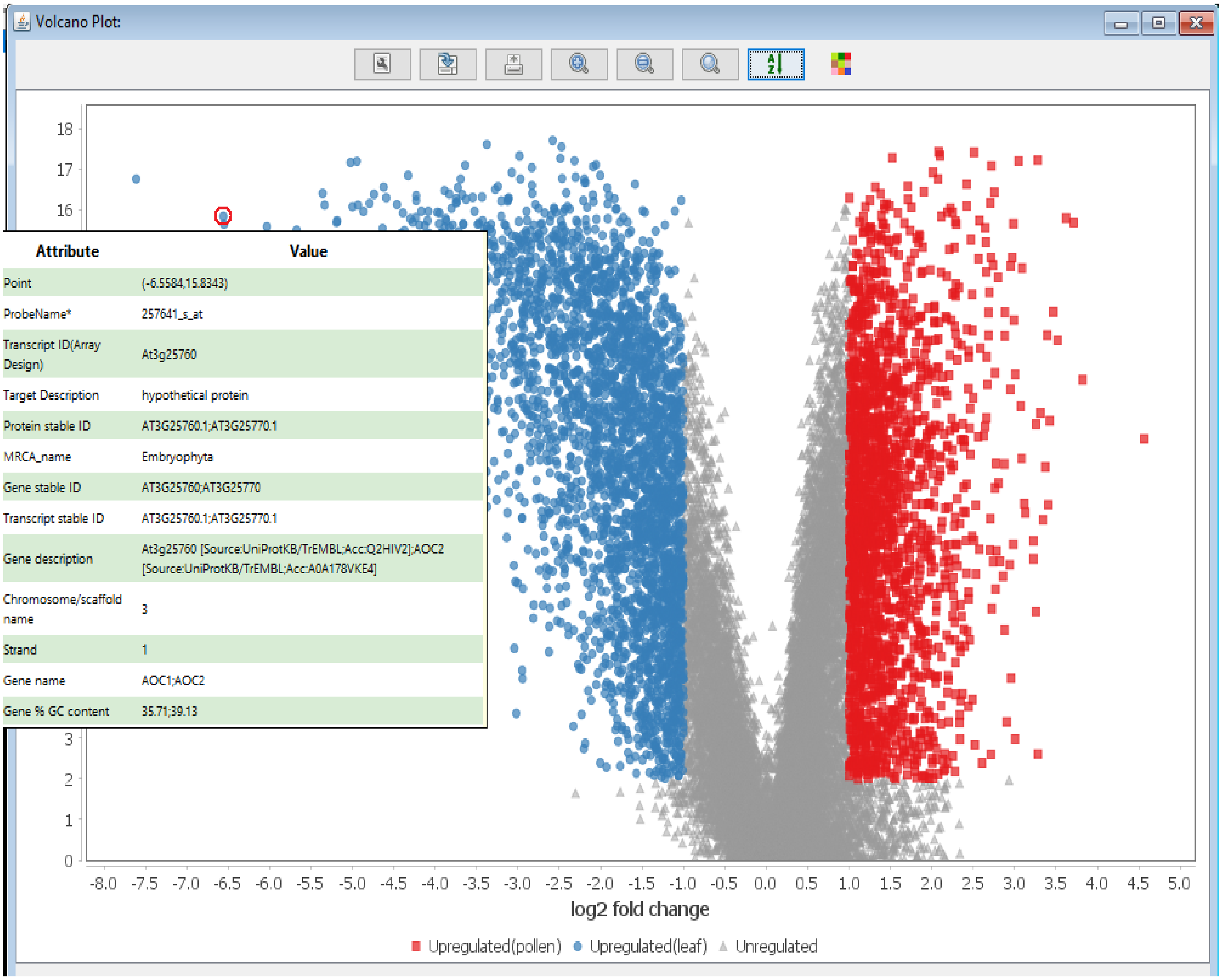

Supplementary Figure 9. Differential expression analysis followed by volcano plot visualization using MOG. Gene metadata, revealed upon hovering the mouse over a data point, shows At3G25760, a gene of Embryophyta (photosynthetic land plants), is about 50-fold more highly accumulated in leaves relative to pollen (Mann-Whitney U test; B-H corrected p-value$< 10^{-16}$. Among these is At3G25760, specific to Arabidopsis and encoding a protein of unknown function; it is about 50-fold upregulated. A Pearson's correlation of this gene versus all genes across all samples, followed by a GO enrichment test of all genes with a correlation greater than 0.70, indicates the most significantly enriched genes encode enzymes of biosynthesis of jasmonic acid, a hormone that orchestrates injury response in plants[3], fungal-responses, and wounding responses.

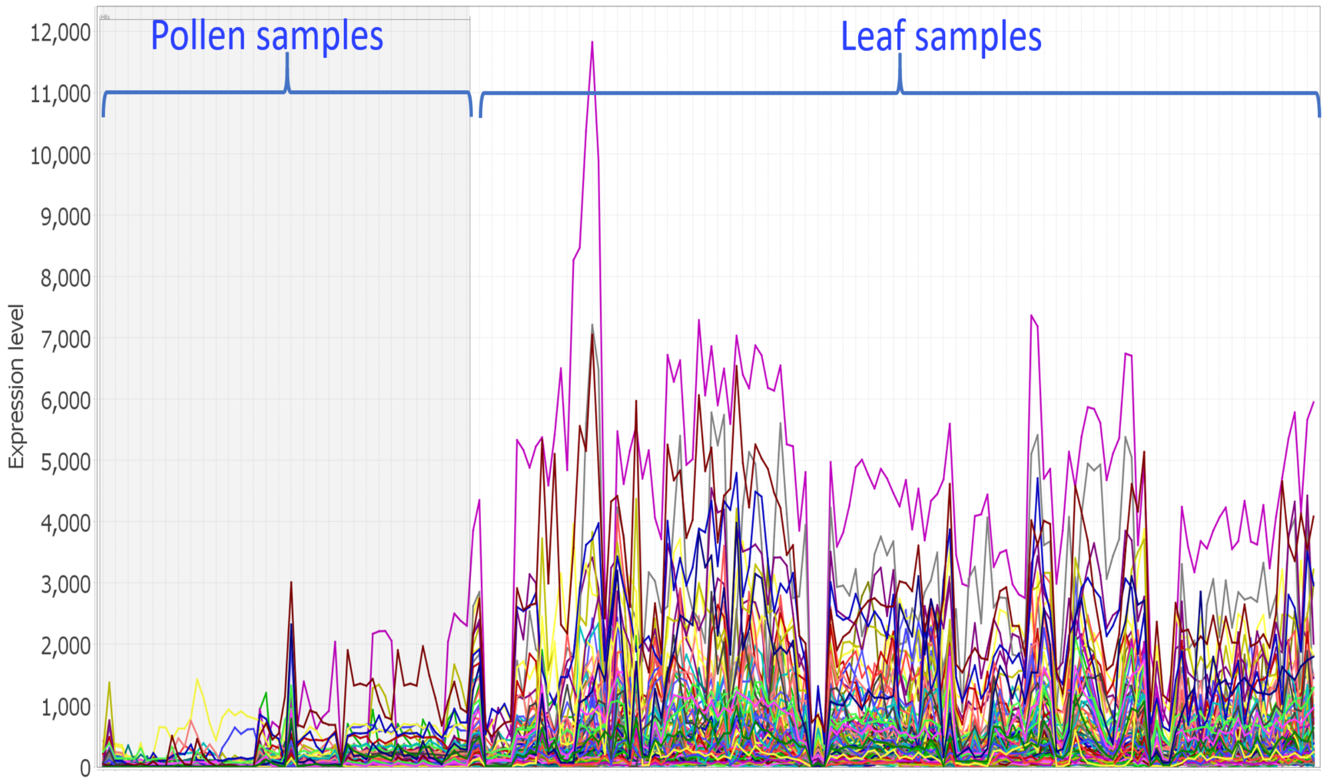

Supplementary Figure 10. Genes coexpressed with AT1G67860 (Spearman correlation > 0.65) are highly expressed in leaf samples as compared to pollen.

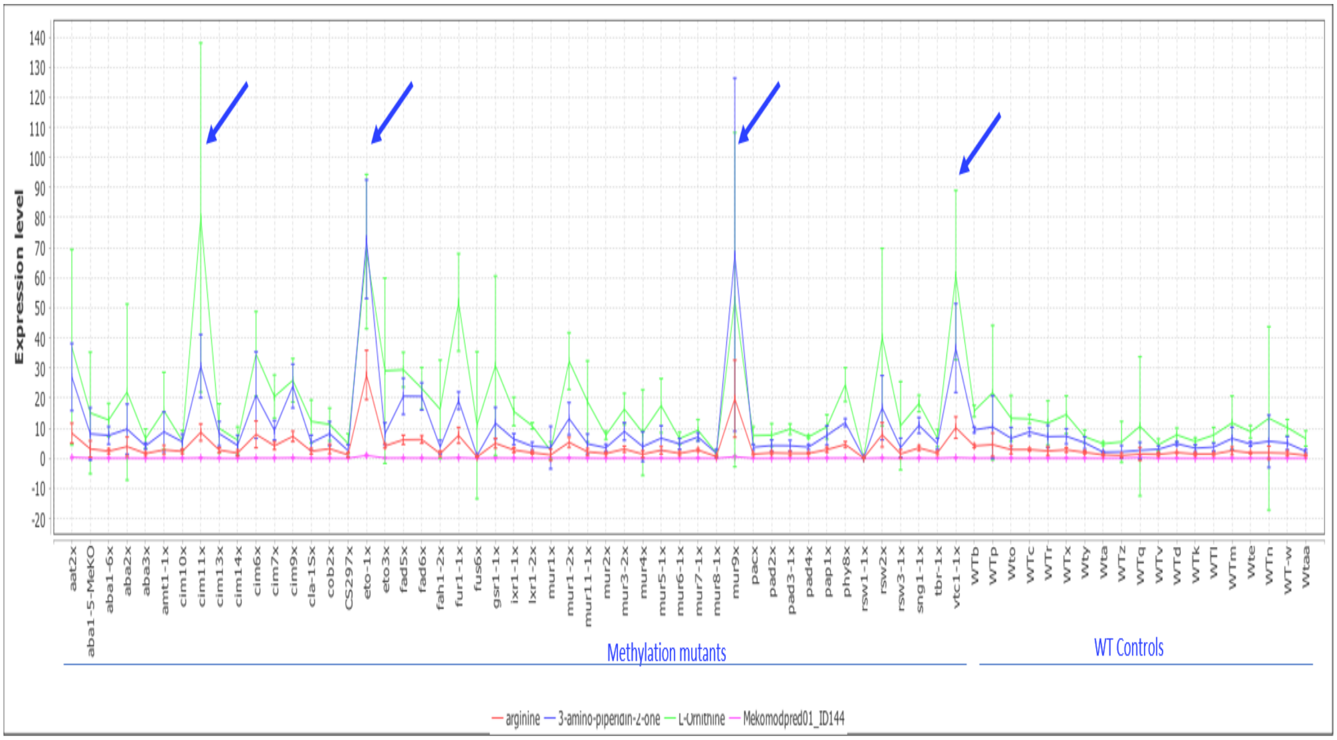

Supplementary Figure 11. Upregulation of correlated metabolites in Arabidopsis. There is a significant increase in accumulation of these metabolites in four methylation mutants. Each data point is means of 8-16 biological replicates. (*transcribed ORFs*) genes. Metabolomics data from[4] downloaded from PMR (http://metnetweb.gdcb.iastate.edu/PMR/).

References

1. Supek, F., Bošnjak, M., Škunca, N. & Šmuc, T. Revigo summarizes and visualizes long lists of gene ontology terms. *PloS one* **6**, e21800 (2011).

2. Shannon, P. *et al.* Cytoscape: a software environment for integrated models of biomolecular interaction networks. *Genome research* **13**, 2498–2504 (2003).

3. Koo, A. J. & Howe, G. A. The wound hormone jasmonate. *Phytochemistry* **70**, 1571–1580 (2009).

4. Fukushima, A. *et al.* Metabolomic characterization of knockout mutants in arabidopsis: development of a metabolite profiling database for knockout mutants in arabidopsis. *Plant physiology* **165**, 948–961 (2014).
