# Supplementary material for: MetaOmGraph: a workbench for interactive exploratory data analysis of large expression datasets
Source: Nucleic Acids Res. 2020 Jan 20;48(4):e23. doi: 10.1093/nar/gkz1209 (PMC7039010; doi:10.1093/nar/gkz1209)
Supplement: gkz1209_Supplemental_Files [file gkz1209_supplemental_files.zip › SupplementaryFile1.pdf]

MetaOmGraph: a workbench for interactive exploratory data analysis of large expression datasets (Supplementary Materials)

Urminder Singh[1,2,3], **Manhoi Hur**[2], **Karin Dorman**[1,3,4], **and Eve Syrkin Wurtele**[1,2,3*]

[1]Bioinformatics and Computational Biology Program, Iowa State University, Ames, IA 50011, USA
[2]Center for Metabolic Biology, Iowa State University, Ames, IA 50011, USA
[3]Department of Genetics Development and Cell Biology, Iowa State University, Ames, IA 50011, USA
[4]Department of Statistics, Iowa State University, Ames, IA 50011, USA
[*]e-mail: mash@iastate.edu

Contents

1 Getting started with MOG. **1**
1.1 Obtaining MOG. 1
1.2 Download a pre-compiled project 1
1.3 Starting MOG 1
2 Creating a new MOG project. **1**
2.1 Expression data files for a new MOG project. 1
2.2 Sample metadata files for a new MOG project. 1
2.3 MOG project identifiers. 1
2.4 Challenges of integrating data from multiple studies. 2
3 Permutation test for significance of association **2**
References **3**

1 Getting started with MOG.

1.1 Obtaining MOG.

MOG is freely available to download from http://metnetweb.gdcb.iastate.edu/MetNet_MetaOmGraph.htm. Click the download button, and then download the .zip file. Unzip the downloaded file to get a .jar file, this is the MOG program. MOG's source code along with a comprehensive user guide is available at https://github.com/urmi-21/MetaOmGraph/.

1.2 Download a pre-compiled project

Select a project from the same page that you download MOG from (http://metnetweb.gdcb.iastate.edu/MetNet_MetaOmGraph.htm) and download it. **Keep each of the three project files (the .mog file, the metadata file, and the data file) in the same directory** (It is OK to add other files to the folder).

1.3 Starting MOG

DOUBLE CLICK on the **.jar** file icon to start the MOG program.

2 Creating a new MOG project.

2.1 Expression data files for a new MOG project.

The *expression data* file contains unique identifiers (IDs) for each feature (e.g, gene) followed by information about that feature and then numerical data quantifying each feature across multiple conditions. For RNA-Seq analyses, each row would include a unique gene ID followed by metadata about that gene and the quantified expression level of that gene over multiple RNA-Seq runs (Supplementary Figure 1). If the data are from metabolomics studies, each row would provide a unique ID for each metabolite, metadata about each metabolite, and numerical data quantifying accumulation of each metabolite over multiple GC-MS analyses. The data columns of expression data files, which contain numerical expression values, are headed by unique IDs for the corresponding samples.

Metadata about each feature can be added or modified by the researcher, simply by adding or modifying columns of the file. (Projects provided on the MOG website can also be modified). For example, for proteomics studies, a researcher might want metadata to include: function, pathway membership, length in amino acids, phylostratum[1], tertiary structure, amino acid content, and PubMed references; for metabolomics studies, metadata might include: InChi key, PubChemID, systematic name, synonyms, and molecular weight.

2.2 Sample metadata files for a new MOG project.

Metadata is input as a delimited file (Supplementary Figure 1). The first column of each row contains a unique sample ID corresponding to the sample IDs present in the data file. Other columns might contain the study ID, a description of the experimental design, experimental protocol, biological material sampled, instrument used, study abstract. Information can be added or modified by the user simply by adding columns to the file. For example, a researcher might wish to add fields for references, dates, notes, or keywords.

A caveat of leveraging the archived metadata is that it is only as good as the metadata provided; metadata about the samples may be incomplete or misleading, and the quality varies from study to study[2,3]. Despite the many limitations in metadata, metadata is key to understanding and interpreting aggregated experiments[2,3].

The metadata for the features and samples adds extra dimensions to data exploration in MOG. For example, researchers can visualize expression of selected genes for selected treatments as compared to the other treatments (e.g., the expression of transcription factors of the NF-Y family in studies about mutants in fatty acid biosynthesis enzymes compared to mutants in enzymes of other pathways). Researchers can also filter metadata to select certain samples (e.g., samples containing the word "kidney" in the "abstract" field, and the words "stage IV" in the "tumor stage" field) for further exploration.

2.3 MOG project identifiers.

To conveniently store and share the MOG projects, for each dataset we have assigned a unique identifier which consists of five fields, i.e. *Species-Description-#Features-#Samples-Type*. For the *species* field we use the initials of

the species name e.g. HU for Human. The *description* field contains one or two word description of the dataset. *#Features* and *#samples* are the number of features and samples present in the dataset. The last field, *type*, describes the type of data i.e. RNA-Seq, microarray, meabolomics, or proteomics.

2.4 Challenges of integrating data from multiple studies.

MOG is designed for exploration of complex datasets composed of multiple samples from different studies. The data supplied to MOG should be comparable across these samples and studies. Thus, appropriate normalization should be performed on the data and effects of unwanted factors should be removed from the data[4–6]. Although the steps of data normalization and integration are not a part of MOG, we present several considerations.

Two predominate frameworks for analysis of multiple expression studies are: 1) analyze each study independently and then combine results from independent studies (meta-analysis)[7–14], or 2) combine data from multiple studies together to create a "pooled" dataset and analyze the pooled data[7,14,15].

In meta-analysis, if individual studies show statistically- significant results, then it is likely that the final result combined from the studies will also be significant[7]. This approach avoids the major challenge of normalizing data across studies[7,14]. A caveat is that the number of samples in an individual study must be high enough to allow statistical inference[7] and a major drawback of this method is that it does not allow to directly compare the expression values across studies.

The pooling approach has the advantage that combining a large number of samples can result in better statistical power and inference; a second advantage is that the expression values can be compared across studies[7,16,17]. The major challenge is data normalization across studies. Despite much research on how to merge large numbers of diverse biological studies together[16,18–21], combining heterogeneous studies, particularly of RNA-Seq data, is still a challenge because many latent unwanted factors induce variability in the data, which makes it hard to directly compare data from different samples[5,6,22–25].

These unwanted factors may be technical effects (e.g., library size, hardware used in sequencing, the protocol used for extraction) or unknown biological effects (e.g., a temperature drop in one of the growth rooms, or a study in which control participants were residing in a smoggy region) which can render the data uninterpretable in relation to its metadata, in the sense that they introduce additional but unknown covariates in the data[5,6,22]. If these unwanted effects are not removed, estimated associations between features after pooling data from multiple studies may be fallacious[13,14,26]. Thus, before pooling expression data from multiple heterogeneous studies, the effects of unwanted factors must be removed.

3 Permutation test for significance of association

MOG speeds up the computation of conducting permutation test by using mutithreading. Algorithm 1 shows the steps involved in performing a permutation test to calculate the significance of measured stastical association by any measure such as Pearson correlation, Spearman correlation or Mutual information.

Algorithm 1 Permutation test for significance of association

$P \leftarrow$ number of *permutations*
$T \leftarrow$ number of *threads*
$X \leftarrow$ expression values of first gene
$Y \leftarrow$ expression values of second gene
$\rho \leftarrow$ association(X,Y)
$extremes \leftarrow 0$
Execute in parallel P **times using** T **threads :**
    $X^* \leftarrow permute\ (X)$
    $\rho^* \leftarrow$ association(X^*,Y)
    if $|\rho^*| \geq |\rho|$ **then**
        $extremes \leftarrow extremes + 1$
end parallel
$p\text{-}value \leftarrow \frac{extremes+1}{P+1}$

References

1. Arendsee, Z. *et al.* phylostratr: a framework for phylostratigraphy. *Bioinformatics* DOI: 10.1093/bioinformatics/btz171 (2019).
2. Bhandary, P., Seetharam, A. S., Arendsee, Z. W., Hur, M. & Wurtele, E. S. Raising orphans from a metadata morass: A researcher's guide to re-use of public'omics data. *Plant Sci.* **267**, 32–47 (2018).
3. Bernstein, M. N., Doan, A. & Dewey, C. N. Metasra: normalized human sample-specific metadata for the sequence read archive. *Bioinformatics* **33**, 2914–2923 (2017).
4. Evans, C., Hardin, J. & Stoebel, D. M. Selecting between-sample rna-seq normalization methods from the perspective of their assumptions. *Briefings bioinformatics* **19**, 776–792 (2017).
5. Leek, J. T. *et al.* Tackling the widespread and critical impact of batch effects in high-throughput data. *Nat. Rev. Genet.* **11**, 733 (2010).
6. Mostafavi, S. *et al.* Normalizing rna-sequencing data by modeling hidden covariates with prior knowledge. *PLoS One* **8**, e68141 (2013).
7. Lazar, C. *et al.* Batch effect removal methods for microarray gene expression data integration: a survey. *Briefings bioinformatics* **14**, 469–490 (2012).
8. Rau, A., Marot, G. & Jaffrézic, F. Differential meta-analysis of rna-seq data from multiple studies. *BMC bioinformatics* **15**, 91 (2014).
9. Rhodes, D. R. *et al.* Large-scale meta-analysis of cancer microarray data identifies common transcriptional profiles of neoplastic transformation and progression. *Proc. Natl. Acad. Sci.* **101**, 9309–9314 (2004).
10. Ma, T. *et al.* Metaomics: analysis pipeline and browser-based software suite for transcriptomic meta-analysis. *Bioinformatics* (2018).
11. Toro-Domínguez, D. *et al.* Imageo: integrative gene expression meta-analysis from geo database. *Bioinformatics* (2018).
12. Tseng, G. C., Ghosh, D. & Feingold, E. Comprehensive literature review and statistical considerations for microarray meta-analysis. *Nucleic acids research* **40**, 3785–3799 (2012).
13. Lagani, V., Karozou, A. D., Gomez-Cabrero, D., Silberberg, G. & Tsamardinos, I. A comparative evaluation of data-merging and meta-analysis methods for reconstructing gene-gene interactions. *BMC bioinformatics* **17**, S194 (2016).

14. Almeida-de Macedo, M. M., Ransom, N., Feng, Y., Hurst, J. & Wurtele, E. S. Comprehensive analysis of correlation coefficients estimated from pooling heterogeneous microarray data. *BMC Bioinforma.* **14**, 214 (2013).

15. Mentzen, W. I. & Wurtele, E. S. Regulon organization of arabidopsis. *BMC plant biology* **8**, 99 (2008).

16. Rudy, J. & Valafar, F. Empirical comparison of cross-platform normalization methods for gene expression data. *BMC bioinformatics* **12**, 467 (2011).

17. Warnat, P., Eils, R. & Brors, B. Cross-platform analysis of cancer microarray data improves gene expression based classification of phenotypes. *BMC bioinformatics* **6**, 265 (2005).

18. Robinson, M. D. & Oshlack, A. A scaling normalization method for differential expression analysis of rna-seq data. *Genome biology* **11**, R25 (2010).

19. Cao, Q. *et al.* Reconstruction of enhancer–target networks in 935 samples of human primary cells, tissues and cell lines. *Nat. genetics* **49**, 1428 (2017).

20. Zang, C. *et al.* High-dimensional genomic data bias correction and data integration using mancie. *Nat. communications* **7**, 11305 (2016).

21. Willforss, J., Chawade, A. & Levander, F. Normalyzerde: Online tool for improved normalization of omics expression data and high-sensitivity differential expression analysis. *J. proteome research* (2018).

22. Peixoto, L. *et al.* How data analysis affects power, reproducibility and biological insight of rna-seq studies in complex datasets. *Nucleic acids research* **43**, 7664–7674 (2015).

23. Schmidt, F. *et al.* An ontology-based method for assessing batch effect adjustment approaches in heterogeneous datasets. *Bioinformatics* **34**, i908–i916 (2018).

24. Chawade, A., Alexandersson, E. & Levander, F. Normalyzer: a tool for rapid evaluation of normalization methods for omics data sets. *J. proteome research* **13**, 3114–3120 (2014).

25. Paulson, J. N. *et al.* Tissue-aware rna-seq processing and normalization for heterogeneous and sparse data. *BMC bioinformatics* **18**, 437 (2017).

26. Vandenbon, A. *et al.* Immuno-navigator, a batch-corrected coexpression database, reveals cell type-specific gene networks in the immune system. *Proc. Natl. Acad. Sci.* **113**, E2393–E2402 (2016).
